# Presentation of the obsolete drug reserpine in three German-language pharmacology textbooks

**DOI:** 10.1007/s00210-023-02877-9

**Published:** 2023-12-16

**Authors:** Nikolas Misera, Roland Seifert

**Affiliations:** Institute of Pharmacology, Hannover Medical School, Carl-Neuberg-Str. 1, D-30625 Hannover, Germany

**Keywords:** Reserpine, Obsolete drugs, Textbook analysis, Prescription analysis, Hypertension guidelines

## Abstract

For several decades, reserpine was used to treat hypertension and, to a limited extent, psychoses. Over time, however, the indication became more and more restricted to the point of obsolescence. This study examines the extent to which textbooks are up to date in their content and oriented towards therapeutic guidelines, using the obsolete drug reserpine as a paradigm. Three German pharmacology textbook series were examined for the coverage of reserpine from 1964-2023: *Allgemeine und Spezielle Pharmakologie und Toxikologie* (Aktories), *Allgemeine und Spezielle Pharmakologie und Toxikologie* (Karow) and *Pharmakologie und Toxikologie* (Lüllmann). We compared the textbook content with data on reserpine prescriptions and hypertension guidelines and analysed the relevance of reserpine in examinations using German federal exam questions by the Institute for medical and pharmaceutical exam questions (IMPP). The textbooks differ conceptually from each other. The indication of reserpine for hypertension has become more restricted over time in all three textbooks, yet they partially show discrepancies with hypertension guidelines. The reserpine prescription figures show a strong decline , and reserpine has not been queried by the IMPP, which underlines the obsolescence of the drug. Overall, our study shows that the presentation of a representative obsolete drug in pharmacology textbooks lags current medical practice. We also unmasked more differences in the presentation of an obsolete drug in standard textbooks than anticipated. In conclusion, the analysis of obsolete drugs in pharmacology textbooks is an informative way of assessing how up-to-date they are.

**Supplementary Information:**

The online version contains supplementary material available at 10.1007/s00210-023-02877-9.

## Introduction

Reserpine is a naturally occurring alkaloid from the Indian snakeroot (*Rauwolfia serpentina*). It can also be obtained from Australian, African and Mexican snakeroot (Giebelmann and von Meyer 2023, https://www.gtfch.org/cms/images/stories/media/tk/tk70_2/Giebelmann2.pdf, last accessed 11 October 2023).

Reserpine was used in the Indian healing art for centuries (Ritter et al. 2019). In 1931, Sen and Bose reported on a good treatment possibility of “insane people” by reserpine. In 1949, Vakils reported the antihypertensive effect of reserpine in the British Heart Journal (Isharwal and Gupta 2006, https://www.ncbi.nlm.nih.gov/pmc/articles/PMC1524711/, last accessed 11 October 2023), and in 1952, reserpine was isolated from snakeroot (Schlitter et al. 1952, 10.1007/BF02174406, last accessed 11 October 2023). In the 1950s, reserpine found particular use in psychiatry but was eventually superseded by the superior chlorpromazine in the treatment of schizophrenia (Kuschinsky and Lüllmann 1964). Reserpine was, however, historically very important for basic research on depression. Due to the ADR of causing depression and its mechanism of action (reduction of catecholamines), it was assumed that depression correlates with a monoamine deficiency in the central nervous system (Aktories et al. 2022).

Reserpine was widely used in the treatment of hypertension in the following decades. Due to its severe adverse effects, its indication was increasingly restricted (Lüllmann and Mohr 1999).

There is the assumption that reserpine would not have fallen into such disrepute if it had been used earlier in lower doses with which an effective lowering of blood pressure would also have been possible. The severe ADRs were consequences of the high doses of reserpine in which it was used previously (Aktories et al. 2017).

Reserpine belongs to the class of antisympathotonics. Its effect is based on the reduction of catecholamines by inhibiting the reuptake of noradrenaline and dopamine by inhibiting the transport function of VMAT2. Thus, it is a reuptake inhibitor (Brunton and Knollmann 2022). The activity of the sympathetic nervous system is consequently reduced. At higher doses, reserpine causes an emptying of catecholamine stores. The effect of reserpine can be divided into a central and peripheral component. Peripherally, it causes in particular a reduction in blood pressure, for which reserpine is also used, but also, for example, the typical ADR of a blocked nose (rhinitis serpentina). Centrally, reserpine has an antipsychotic effect. Particularly sedation, the parkinsonoid effect and depression are to be mentioned as ADRs (Lüllmann and Mohr 1999).

Textbooks are used by many medical students to prepare for exams. To be able to practice according to modern evidence-based treatment concepts after graduation, it is important that the teaching is oriented towards guidelines as much as possible.

**Fig. 1:**
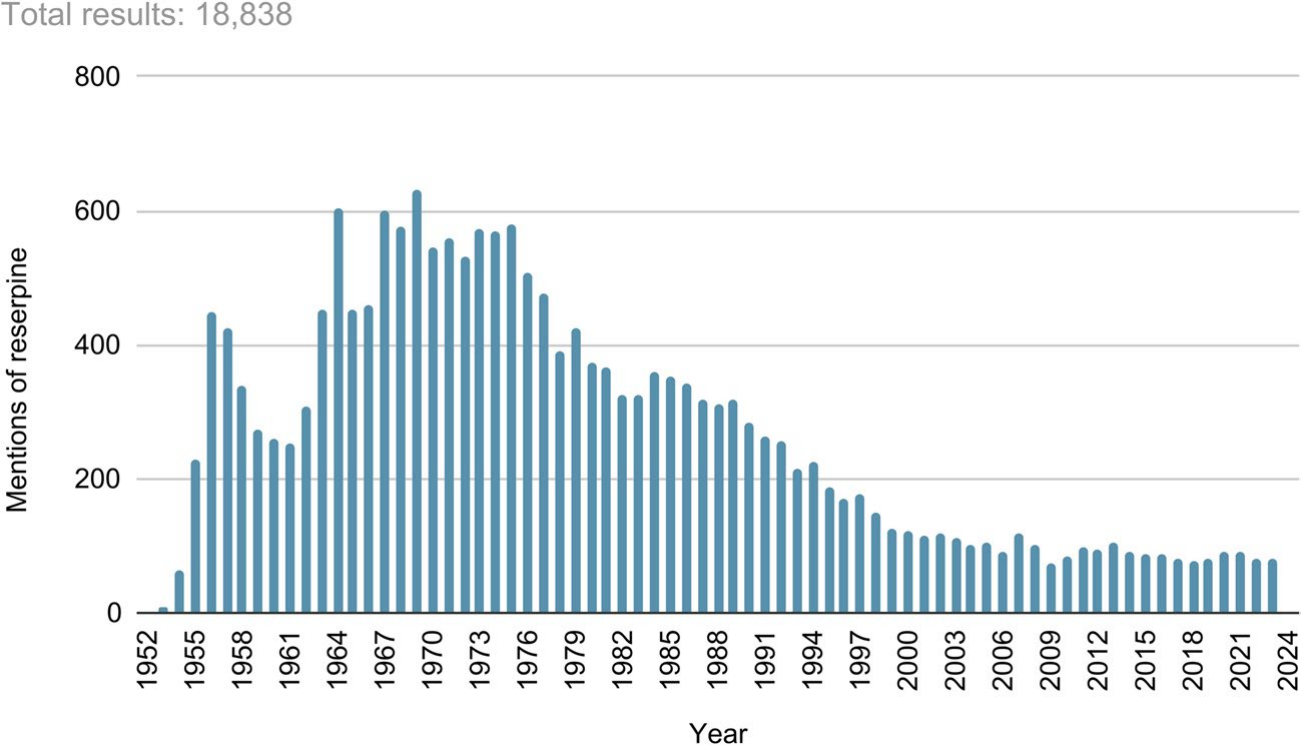
Results in PubMed regarding reserpine: the chart shows the total number of reserpine mentions in PubMed, broken down by year

**Fig. 2:**
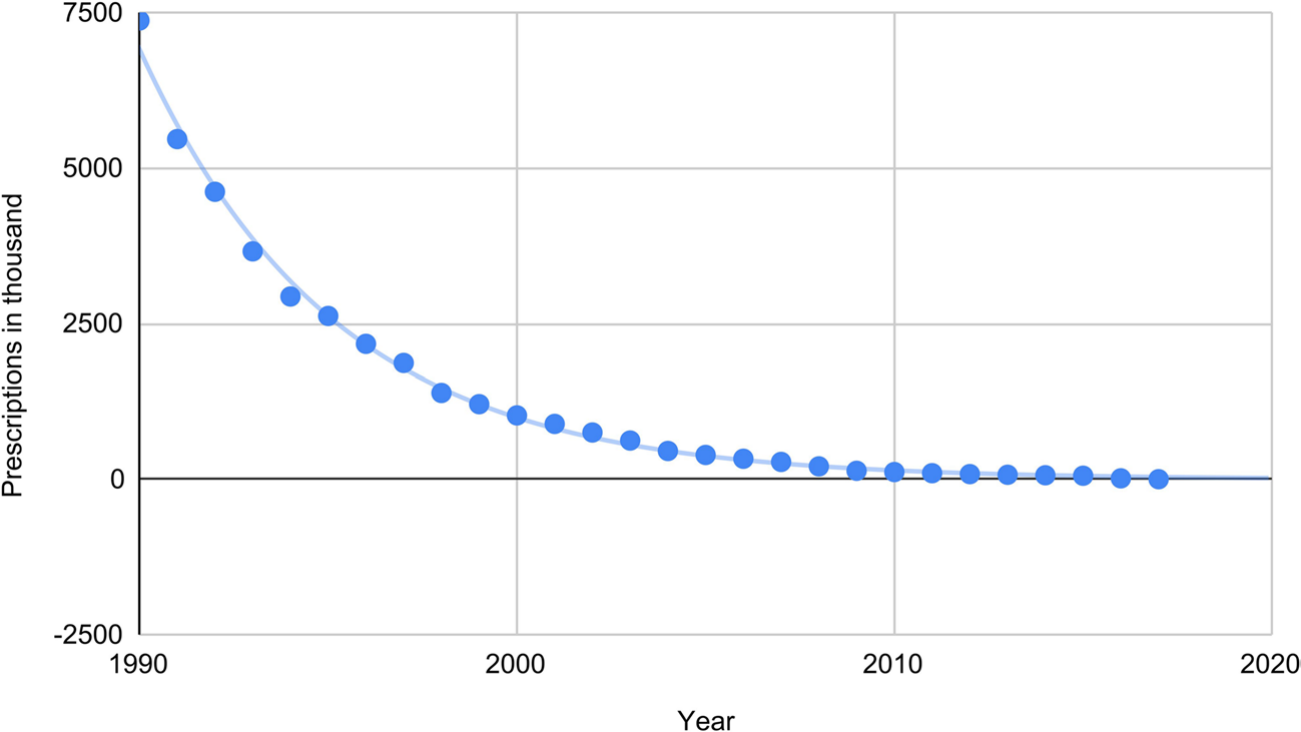
Prescription numbers of reserpine from 1990 onwards described by an E-function: the prescription figures regarding reserpine can be approximately described by an E-function. The data were provided by the WIdO and contain information regarding prescriptions of reserpine from 1990 to 2017 in Germany

As a search in PubMed on reserpine shows (Fig. 1), the search results have decreased continuously since the 1970s. In addition, the decline in prescription numbers is also interesting to mention here. The Wissenschaftliches Institut der Ortskrankenkassen (WIdO) has been registering prescription figures as well as costs for drugs in Germany for decades. Thus, especially on the German market, textbooks can be compared well with the relevance in therapy. This was also a reason for the selection of German textbooks for the analysis. As Fig. 2 shows, the decline in reserpine prescriptions from 1990 onwards can be described approximately with an E-function.

The decrease in the relevance of reserpine in teaching is also visible in the absence of any IMPP questions on reserpine. The Institute for Medical and Pharmaceutical Examination Questions (IMPP) is responsible for the design of examination questions in the written part of the state examinations in the study of human medicine. The IMPP questions were accessed from the online learning programme Viamedici.

Collectively, prescription figures, IMPP questions and a cursory analysis in PubMed show a sharp decline in the relevance of reserpine. Nowadays, it is only of limited importance. It is interesting to analyse whether this development is also visible in textbooks or whether there are other reasons for a more extensive mention of reserpine in textbooks.

The present work examines the topicality of textbooks and the extent to which they are oriented towards modern therapy concepts and guidelines, using a drug as an example. To the best of our knowledge, our work is one of the first to analyse textbooks for the development of the presentation of an obsolete drug. The analysis of the historical development of pharmacology textbooks is of great importance so that they are up-to-date and reflect current therapeutic guidelines and medical practise.

## Materials and methods

In order to analyse the extent to which textbook content changes over time, three textbooks were selected: *Allgemeine und Spezielle Pharmakologie und Toxikologie* (Aktories), *Allgemeine und Spezielle Pharmakologie und Toxikologie* (Karow) and *Pharmakologie und Toxikologie* (Lüllmann).

The textbook Karow currently has 31 editions, which are published annually. The first edition appeared in 1993 (Karow and Lang 1993). Lüllmann and Aktories have longer and more irregular publication intervals. Lüllmann is the oldest of the three textbooks; its first edition was published in 1964 (Kuschinsky and Lüllmann 1964). The current edition (18th edition) appeared in 2016 (Lüllmann et al. 2016). The first edition of Aktories was published 11 years later (Forth et al. 1975), and the current edition (13th edition) was published in 2022 (Aktories et al. 2022). Editions five and eight of Karow could not be analysed due to procurement problems, but since the pages concerning reserpine in editions four (Karow and Lang 1996) and six (Karow and Lang 1998) as well as seven (Karow and Lang 1999) and nine (Karow and Lang 2001) are identical and the differences between the editions are only marginal, this should have no influence on the results. For better illustration, it was assumed in diagrams that the two editions are identical with their previous and following edition. Table 1 shows an overview of all editions of the corresponding textbooks.

**Table 1:** The various editions of the textbooks. The table shows the pages relating to reserpine in the editions of the three textbook series. If a page is listed in the keyword index but reserpine is not mentioned on the corresponding page, this page is not listed here. Pages designated as continuing pages in the index or subsequent pages to which information on reserpine is continued are listed here as individual pages. The year of publication for Karow always shows the year to which the textbooks refer with its cover, even if the edition was published the previous year

Textbook series	Edition	Reference	Reserpine related pages (keyword index)
Aktories	1	Forth et al. (1975)	125, 126, 129, 404, 409, 435
Aktories	2	Forth et al. (1977)	119, 125, 413, 417, 422
Aktories	3	Forth et al. (1980)	57, 119, 125, 413, 417, 422
Aktories	4	Forth et al. (1983)	57, 136, 137, 138, 140, 147, 148, 149, 457, 461, 467, 493
Aktories	5	Forth et al. (1987)	63, 150, 151, 152, 159, 161, 508, 513, 519, 549
Aktories	6	Forth et al. (1992)	108,180, 181, 190, 275, 278, 279, 379, 449
Aktories	7	Forth et al. (1996)	100, 106, 111, 116, 166, 173, 187, 188, 189, 279, 297, 428, 431, 595
Aktories	8	Forth et al. (2001)	107, 115, 126, 180, 189, 205, 206, 207, 221, 348, 505, 509, 686
Aktories	9	Aktories et al. (2004)	109, 119, 128, 177, 186, 200, 201, 202, 476
Aktories	10	Aktories et al. (2009)	109, 117, 162, 173, 187, 188, 189, 190, 479
Aktories	11	Aktories et al. (2013)	99, 106, 112, 154, 163, 178, 179, 180, 285, 465
Aktories	12	Aktories et al. (2017)	86, 89, 95, 99, 138, 147, 161, 162, 263, 265, 433
Aktories	13	Aktories et al. (2022)	97, 99, 105, 108, 150, 159, 161, 163
Lülllmann	1	Kuschinsky and Lüllmann (1964)	26, 34, 35, 36, 163
Lüllmann	2	Kuschinsky and Lüllmann (1966)	27, 35, 36, 166
Lüllmann	3	Kuschinsky and Lüllmann (1969)	2, 26, 27, 34, 35, 36, 49, 167
Lüllmann	4	Kuschinsky and Lüllmann (1970)	3, 28, 36, 37, 51, 176
Lüllmann	5	Kuschinsky and Lüllmann (1971)	3, 28, 36, 37, 38, 51, 179
Lüllmann	6	Kuschinsky and Lüllmann (1974)	3, 25, 26, 32, 33, 45, 156, 300
Lüllmann	7	Kuschinsky and Lüllmann (1976)	65, 69, 73, 74, 89, 92, 210, 219
Lüllmann	8	Kuschinsky and Lüllmann (1978)	71, 75, 82, 83, 98, 101, 241
Lüllmann	9	Kuschinsky and Lüllmann (1981)	47, 72, 77, 85, 86, 103, 107, 248, 419
Lüllmann	10	Kuschinsky and Lüllmann (1984)	56, 83, 89, 99, 100, 121, 126, 290, 486
Lüllmann	11	Kuschinsky and Lüllmann (1987)	86, 103, 104, 105, 132, 253, 341, 544
Lüllmann	12	Kuschinsky and Lüllmann (1989)	94, 105, 106, 135, 136, 560
Lüllmann	13	Kuschinsky et al. (1993)	97, 102, 112, 113, 126, 158
Lüllmann	14	Lüllmann and Mohr (1999)	76, 87, 88, 127, 522
Lüllmann	15	Lüllmann et al. (2002)	79, 90, 91, 545
Lüllmann	16	Lüllmann et al. (2006)	102
Lüllmann	17	Lüllmann et al. (2015)	52, 104, 114, 115
Lüllmann	18	Lüllmann et al. (2016)	74, 139, 140
Karow	1	Karow and Lang (1993)	70, 71, 140, 359
Karow	2	Karow and Lang (1994)	70, 71, 140, 359
Karow	3	Karow and Lang (1995)	70, 71, 140, 359
Karow	4	Karow and Lang (1996)	70, 71, 140, 359
Karow	6	Karow and Lang (1998)	70, 71, 140, 359
Karow	7	Karow and Lang (1999)	78, 79, 163, 455
Karow	9	Karow and Lang (2001)	78, 79, 165, 477
Karow	10	Karow and Lang (2002)	78, 79, 165, 477
Karow	11	Karow and Lang-Roth (2003)	78, 79, 495
Karow	12	Karow and Lang-Roth (2004)	91, 92, 608
Karow	13	Karow and Lang-Roth (2005)	92, 93, 652
Karow	14	Karow and Lang-Roth (2006)	136, 137, 673
Karow	15	Karow and Lang-Roth (2007)	136, 137, 693
Karow	16	Karow and Lang-Roth (2008)	136, 137, 704
Karow	17	Karow and Lang-Roth (2009)	134, 135, 714
Karow	18	Karow and Lang-Roth (2010)	13, 144, 145, 748
Karow	19	Karow and Lang-Roth (2011)	11, 127, 128, 706
Karow	20	Karow and Lang-Roth (2012)	19, 135, 136, 714
Karow	21	Karow and Lang-Roth (2013)	19, 137, 138, 724
Karow	22	Karow and Lang-Roth (2014)	19, 135, 136, 722
Karow	23	Karow and Lang-Roth (2015)	19, 135, 136, 720
Karow	24	Karow and Lang-Roth (2016)	19, 135, 136, 750
Karow	25	Karow and Lang-Roth (2017)	19, 135, 136, 752
Karow	26	Karow and Lang-Roth (2018)	19, 134, 135, 764
Karow	27	Karow and Lang-Roth (2019)	19, 134, 135, 772
Karow	28	Karow and Lang-Roth (2020)	19, 134, 135, 768
Karow	29	Karow and Lang-Roth (2021)	19, 134, 135, 761
Karow	30	Karow and Lang-Roth (2022)	19, 134, 135, 763
Karow	31	Karow and Lang-Roth (2023)	19, 130, 131, 759

Criteria for analysis were established in advance, as well as a coding for the criteria. Only the listings on reserpine in the keyword index of the textbooks were used as the basis for the analysis. Thus, different criteria were designed to compare the textbooks with each other as well as the individual editions.

### Extent and mentions of reserpine in the textbooks

In order to show the extent to which reserpine is mentioned in the textbooks, various criteria were designed. The number of mentions of reserpine in the keyword index was recorded.

In addition, the text length in which reserpine is discussed was analysed. A code was designed: code 1, only mention (in chart, table or text); code 2, short paragraph (up to 5 lines); code 3, long paragraph (6–10 lines); code 4, up to half a page and code 5, up to one page.

Since the textbooks differ in their formatting among themselves as well as between the textbook series, the coding was again converted into lines in order to compare them better: code 1, 3 lines; code 2, 5 lines; code 3, 10 lines; code 4, 37 lines and code 5, 83 lines. This calculation is based on the assumption that an average page consists of 110 lines and thus a code 4 corresponds on average to ${~}^{1}\!\left/ \!{~}_{3}\right.$ of a page (37 lines) and code 5 to ¾ of a page (83 lines).

### Thematic analysis in the textbooks

For the thematic analysis, it was first analysed in which chapters reserpine is mentioned. For this purpose, the chapter names were divided into five chapter groups due to their diversity:

1. Nervous system/antisympathotonics: antisympathotonics/pharmacology of the noradrenergic and adrenergic system; basics of pharmacology of the nervous system; sympathomimetics; autonomic organs and sympathetic system.

2. Cardiovascular system, treatment of hypertension: treatment of hypertension; pharmacotherapy of heart failure; substances to reduce blood clotting; pharmacology of the cardiovascular system: the blood vessels; treatment of hypotension and orthostatic dysregulation.

3. Psychotropic drugs: antiepileptic drugs; antiparkinsonian drugs; psychotropic drugs: neuroleptics, antipsychotics; antidepressants and lithium.

4. Neurotransmitters, e.g. de-storage and release of serotonin: neurotransmitters; hypothalamic and pituitary hormones; 5-HT receptor agonists; biogenic amines.

5. Data, etc: pharmacokinetics; time table; harm to the foetus and embryo by drugs.

In the following, it was analysed how many entries occur in the different chapter groups on reserpine per edition. The individual entries on reserpine can be described by a keyword (a main topic). The number of entries for a keyword was analysed. In addition, the number of different topics concerning reserpine that are addressed in an edition was surveyed.

Furthermore, it was examined how often the different topics occur in a textbook overall, if all editions of a textbook are considered together. In this way, it was possible to compare which topics are common to the three textbook series and which only occur in two or even only in one textbook series.

Lastly, it was analysed which are the most frequent topics in Aktories and Lüllmann and in what percentage of the editions they occur. A frequent topic is one that occurs in at least one of the textbook series (Aktories and Lüllmann) in at least 50% of the editions.

### Analysis of the presentation, ADRs, changes to previous editions, dosages and trade names in the textbooks

The didactic presentation is accomplished with diagrams, tables or structural formulae. These three different elements are referred as graphic presentations here. For the three textbooks, it was analysed how many diagrams, tables and structural formulae are shown and how this changes over time between the editions. It was also analysed how the ratio of graphic presentations to the number of entries per edition behaves. In addition, it was studied how the ratio of charts among the graphic presentations in Lüllmann and Aktories behaves.

For the ADRs, the number of ADRs mentioned was recorded per edition and textbook series. In addition, it was analysed which ADRs were mentioned and to which textbook series they were common to and which were possibly only reserved for individual textbook series.

To analyse how an edition changes from the previous edition, a code was designed. Per entry, it was analysed whether changes occur. Depending on how extensive the changes are, they were divided into four groups. (1) completely new entry, (2) extensively revised entry, (3) small change in wording and (4) no change or only changes in design.

All references to dosages of the textbooks were compared with each other to show possible discrepancies.

It was identified which trade names are mentioned in which editions of the textbooks. In addition, the trade names were analysed for their linguistic peculiarities.

### Analysis of the indications for reserpine in the textbooks

The strength of the indications up to obsolescence was also analysed. To compare the given indications with each other, first, the strength of the indications mentioned was compared. For the strength of indications, all different editions of the three textbooks were compared with each other and ranked from weak to strong indication (see Appendix 5 and 6). Several editions can also occupy one “place”. The higher the number of the coding, the stronger the indication compared to the other editions. The codes of the strength of indication for hypertension treatment can be described as follows:

1. Application is not described.
2. Obsolete, but still used.
3. Obsolete, but use is described in more detail.
4. Reserpine is a drug of further choice; the ADRs are described as very severe.
5. The ADRs described in the past are due to too high doses. The advantages of reserpine are described, but no more detailed application is explained.
6. Indication for therapy-refractory hypertension, severe forms of hypertension and in case of existing contraindication to beta-blockers. Therapeutic use is limited to severe, otherwise resistant forms of hypertension because of the ADRs.
7. Indication for therapy-refractory hypertension, severe forms of hypertension and with existing contraindication for beta-blockers. Therapeutic use is significantly limited due to the ADRs.
8. Indication for therapy-refractory hypertension, moderate forms of hypertension and with existing contraindication for beta-blockers. The therapeutic applicability is clearly limited due to the ADRs.
9. Due to the side effects, an indication is only given for severe forms of hypertension.
10. Indication for hypertension treatment. The ADRs are weighted more heavily.
11. Indication for hypertension treatment. Reserpine is described as well effective.

The codes of indication strength for the treatment of psychoses can be described as follows:

1. No mention of reserpine for the treatment of psychoses.
2. Reserpine was used in psychiatry in the past. The indication was abandoned due to the severe ADRs.
3. Reserpine was used in psychiatry in the past. However, the reason (severe ADRs) is not mentioned.
4. Reserpine is now only rarely used in psychiatry.
5. There is an indication for the treatment of psychosis with reserpine. However, other drugs are preferred.

### Analysis of prescription numbers of reserpine

In order to show what position reserpine had in treatment, data on prescriptions recorded by the Wissenschaftliches Institut der AOK (WIdO) since 1985 were analysed. The data were requested from the WIdO and examined. It was analysed how the number of prescriptions per year and ATC code changed. Drugs are divided into different groups according to the organ system they affect, chemical, pharmacological and therapeutic aspects. This is described by the ATC code (anatomical - therapeutic - chemical classification). In addition, it was analysed how many different standard aggregate names containing reserpine were prescribed per year, broken down by ATC code, as well as which were the five drugs with the highest prescription per year. In addition, the development of gross costs differentiated by ATC code was presented. A defined daily dose (DDD) is assigned to the active substances. It was analysed which standard aggregate names were prescribed per ATC code and how high the gross costs per DDD per standard aggregate name are.

### Analysis of hypertension guidelines concerning the use of reserpine

Suitable guidelines for the treatment of hypertension were found through an internet search and enquiry at the “Arzneimittelkomission der deutschen Ärzteschaft (AkdÄ)”, “deutschen Hochdruckliga e.V.” and “Arbeitsgemeinschaft der Wissenschaftlichen Medizinischen Fachgesellschaften e.V. (AWMF)". The following guidelines were examined:

- “Arterial Hypertension” of the World Health Organization (WHO) (1978) (https://iris.who.int/bitstream/handle/10665/41632/WHO_TRS_628.pdf?isAllowed=y&sequence=1, last accessed 11 October 2023)
- “Arterielle Hypertonie” of Deutsche Hypertonie Gesellschaft/Deutsche Liga zur Bekämpfung des hohen Blutdrucks (1998) (https://web.archive.org/web/20010412023321fw_/http://www.uni-duesseldorf.de/awmf/ll/index.html, last accessed 11 October 2023)
- “Leitlinien für die Prävention, Erkennung, Diagnostik und Therapie der arteriellen Hypertonie” of Deutsche Liga zur Bekämpfung des hohen Blutdruckes e.V. (Deutsche Hochdruckliga) (2003) (https://web.archive.org/web/20031219180453/http://leitlinien.net/, last accessed 11 October 2023)
- “Arterielle Hypertonie” 2nd edition of Arzneimittelkommission der deutschen Ärzteschaft (2004) (https://www.reanitrain.de/downloads/leitlinien/Notfaelle/internistische%20Notfaelle/zirkulatorische%20Notfaelle/Hypertonie%20-%20hypertensive%20Krise/Arterielle%20Hypertonie.pdf, last accessed 11 October 2023)
- “Pocket Guidelines Management der arteriellen Hypertonie” of Deutsche Hochdruckliga, Deutsche Gesellschaft für Kardiologie, European Society of Cardiology and European Society of Hypertension (2014 and 2018) (https://www.hochdruckliga.de/fileadmin/downloads/mediziner/leitlinien/Pocket-Leitlinie_Hypertonie_2014.pdf, last accessed 11 October 2023), https://leitlinien.dgk.org/files/28_2018_pocket_leitlinien_arterielle_hypertonie_aktualisiert.pdf, last accessed 11 October 2023),
- “Guideline for the pharmacological treatment of hypertension in adults” of the World Health Organization (WHO) (2021) (https://iris.who.int/bitstream/handle/10665/344424/9789240033986-eng.pdf?sequence=1, last accessed October 11, 2023),
- “Global Hypertension Practice Guidelines” of the International Society of Hypertension (2020) (https://www.ahajournals.org/doi/epub/10.1161/HYPERTENSIONAHA.120.15026, last accessed 11 October 2023)

The guidelines were analysed for mentions of reserpine and the presentation and strength of recommendations for treatment with reserpine.

### Analysis of IMPP questions, online learning programmes, PubMed and English textbooks

Textbooks are mainly used to prepare for examinations. Therefore, the extent to which reserpine was examined by the IMPP was analysed. The online learning programme Viamedici was used as the basis for this (https://viamedici.thieme.de/, last accessed 28 July 2023).

Alongside Viamedici, the online learning programme Amboss was also analysed regarding reserpine (https://www.amboss.com/de/, last accessed November 8, 2023).

In addition, it was studied to what extent the number of mentions of content on reserpine in PubMed changes over time. Furthermore, three current reviews and the current editions of three English-language pharmacology textbooks were analysed: *Rang & Dale’s Pharmacology* (Ritter et al. 2019), *Basic & Clinical Pharmacology* (Katzung 2017) and *Goodman & Gilman’s The Pharmacological Basis of Therapeutics* (Brunton and Knollmann 2022).

## Results and discussion

### Extent and mentions of reserpine in the textbooks

**Fig. 3:**
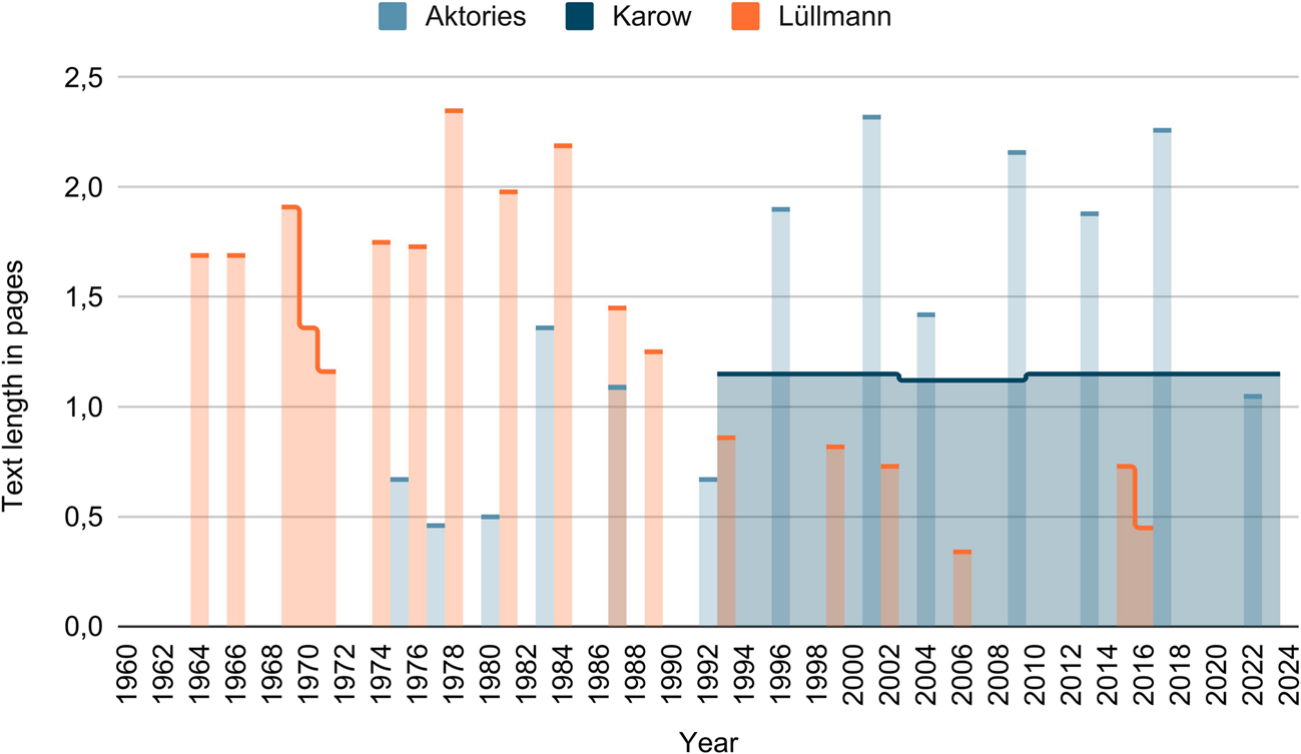
“Text length” on reserpine: the chart shows the “text length” per edition in which reserpine is discussed in the textbooks (Lüllmann, Aktories, Karow). The data was obtained by using the coding explained under methods

Figure 3 shows the text length on reserpine per edition. The text length is given in page numbers. In case of the Aktories, an increase in text length from about 0.5 pages at the beginning to over 2 pages in the penultimate edition wasobserved. A decrease from 1983 to 1992 was noted, with a large increase again in the 1996 edition. It is also interesting to mention the decrease to just over one page in the current edition.

In comparison, a contrasting development was observed in Lüllmann. Although an increase to almost 2.5 pages can be observed in 1978 (starting from just over 1.5 pages in the first edition of 1964), the text length decreased in the following years. In 2016, for example, the text length is less than 0.5 pages.

In Karow, little difference is seen between the individual editions. The text length is just over one page. A slight decrease can be observed in 2003, but the text length increases again to the previous value in 2010.

Figure S1 shows the number of entries in the keyword index for reserpine per edition. Similar developments can be seen here as in Fig. 3. In Lüllmann, for example, an increase to four entries by 1984 can be observed, starting from three entries in 1964. This is followed by a decrease to two entries in the current edition of 2016. 

Furthermore, it is worth mentioning that although there are fewer entries on reserpine in the index in Karow than in the other two textbook series, Karow does not differ here in terms of text length. This underlines the thesis that the information in Karow is given in a more compact way.

### Thematic analysis

The chapter names were divided into different chapter groups. In all three textbooks, most entries are in the chapter group “nervous system/antisympathotonics”. Figure S2 shows this development in Aktories. Actually, the number of entries in this chapter group increases over time. For example, in the first edition there was only one entry in this chapter group, whereas in the current edition, there are even eight entries. This is the highest number of entries on “nervous system/antisympathotonics” of all editions. On the other hand, the entries in the other chapter groups decrease over time. For example, in the 5th edition, there are four entries on “Psychotropic drugs”, and in the 6th edition, there are three entries on “Cardiovascular system, treatment of hypertension” (these two editions are the high points of the corresponding chapter groups), whereas in the current 13th edition, there are no more entries in these chapters. The same applies to the two other chapter groups “Data etc.” (maximum of one entry per edition) and “Neurotransmitters e.g. de-storage and release of serotonin” (maximum of two entries per edition).

The entries on reserpine can be described with a keyword, i.e. a main topic. Figure S3 shows this development in Lüllmann. The first item that stands out is that the largest proportion of each edition can be described with the keyword “mechanism of action”, i.e. inhibition of vesicular dopamine, noradrenaline and adrenaline transport. For example, in the 3rd, 6th, 9th and 10th editions, four entries each, and thus at least half of the entries in an edition, can be described using this keyword. Other entries can be described with the following keywords: “Blockade of the 5-HT transporter of the salivary vesicles by reserpine”, “Application in psychiatry”, “Timeline” and “Foetal damage: Foetal bradycardia and neonatal lethargy”. However, there is no more than a maximum of one entry per edition on this main topic.

**Fig. 4:**
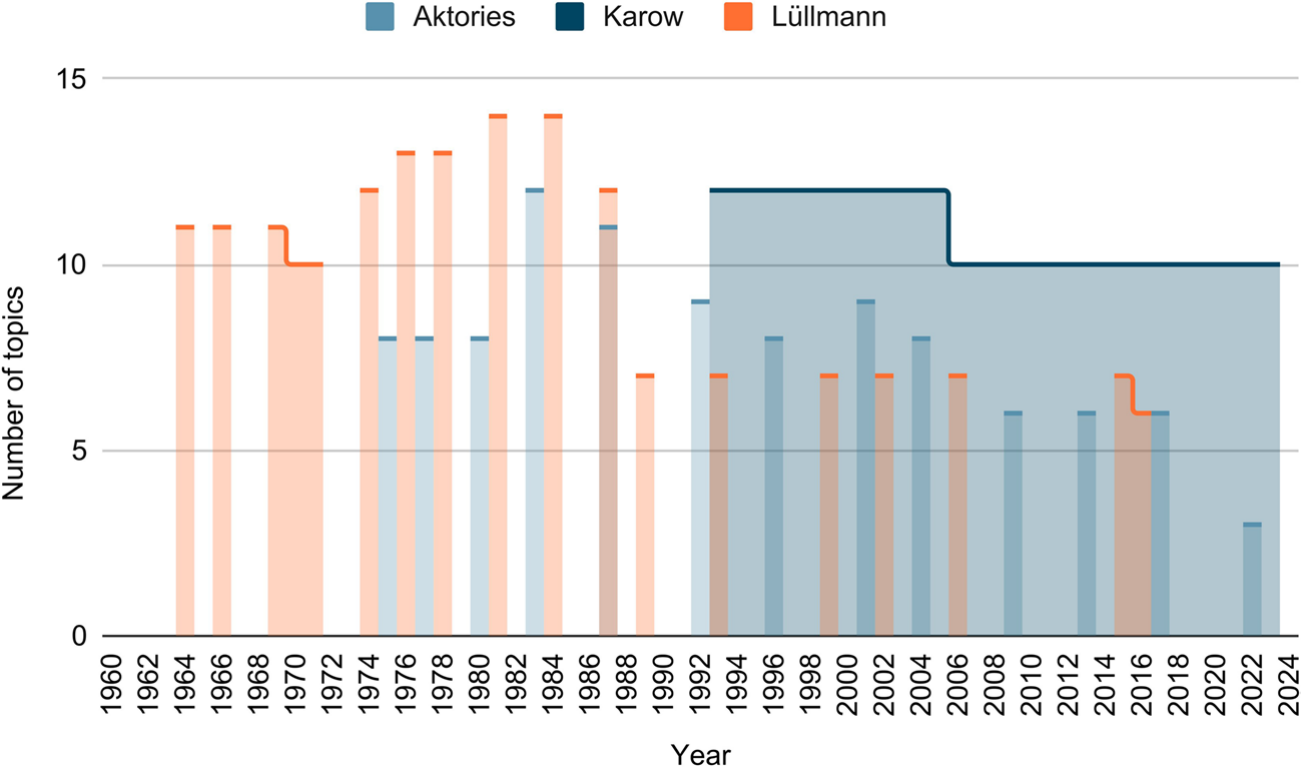
Number of topics: the chart shows how many different topics regarding reserpine are addressed per edition in the three textbooks (Lüllmann, Aktories, Karow)

In addition to the number of entries on a particular topic, it is also interesting to see which other topics are addressed in addition to a main topic. Figure 4 shows the number of topics in the textbooks per edition. In Aktories, an increase can be observed in 1983 (4th edition: 12 topics), followed by a decrease up to the current edition. Lüllmann usually covers more topics per edition than Aktories. The peak is reached with 14 different topics in the 9th (1981) and 10th editions (1984). Here, too, the number of topics decreases in the following editions, down to six topics in the current edition (2016). Karow addresses a similar number of topics per edition as Lüllmann. Again, there are only a few differences between the editions. Only a decrease from 12 to 10 topics from the 13th (2005) to the 14th edition (2006) can be observed, and even in the current edition (2023), with 10 topics, more topics are addressed than in the current editions of the other two textbooks.

**Fig. 5:**
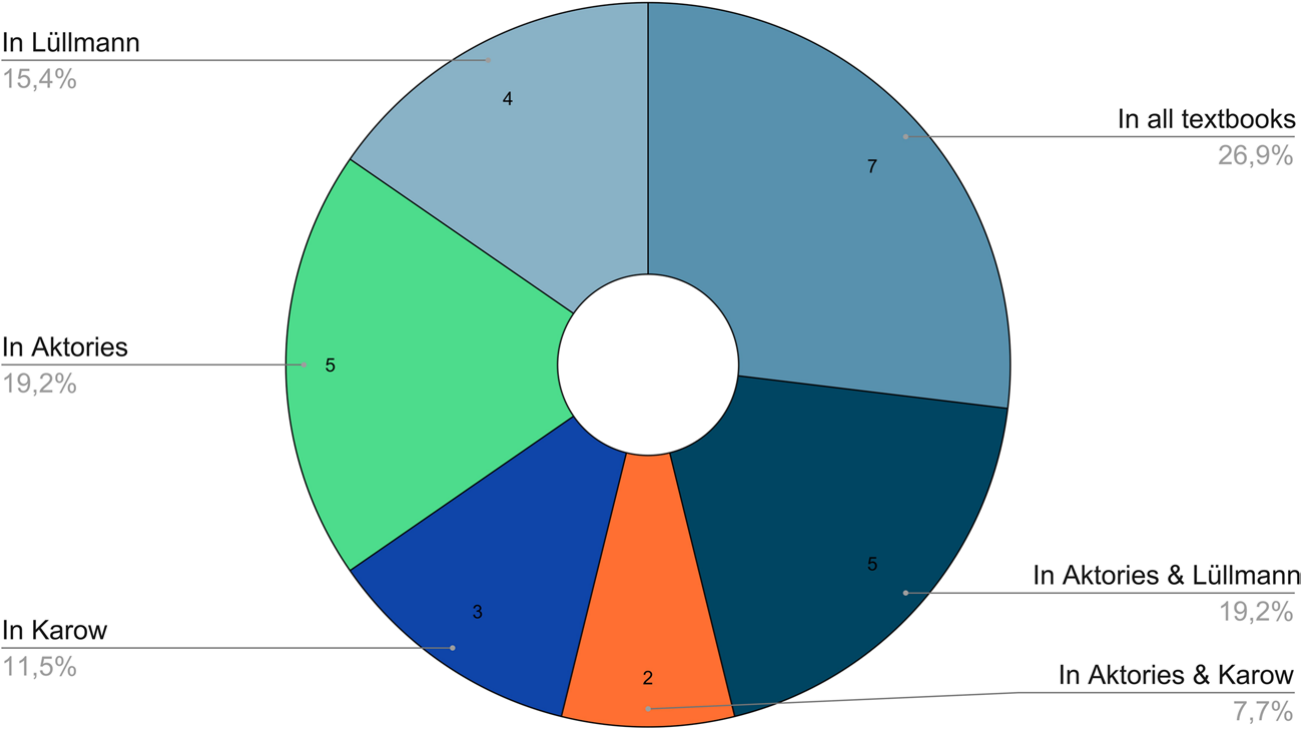
Topics in the various textbooks: the chart shows how many topics regarding reserpine are common to all textbooks (Lüllmann, Aktories, Karow) or how many occur only in one or two different textbook series. Topics common to all textbooks are mechanism of action (inhibition of vesicular dopamine, norepinephrine and epinephrine transport), drug-induced Parkinson’s syndrome, treatment of hypertension, drugs that induce hypotonic circulatory regulation disorders, sympathomimetic reactions at high doses, sedative effect and long-lasting reserpine effect due to irreversible damage to the granules. Topics unique to Aktories: interaction with cardiac glycosides, interfering with coumarin derivatives, reversal of reserpine effect (reserpine reversal), inhibition of vasopressin and oxytocin secretion, no rebound effects after discontinuation of reserpine. Topics unique to Lüllmann: aggressiveness of animals and mentally ill patients is reduced, timeline, substance properties (apolar), foetal damage: foetal bradycardia and lethargy of the newborn child. Topics unique to Karow: interaction with pretreatment with MAO inhibitors, increase in blood pressure, no indication as a sleeping pill or sedative, alcohol consumption as a contraindication

We analysed how the topics are distributed among the different textbook series when all editions of a series are taken together. Figure 5 shows that with seven topics, just over a quarter of all topics relating to reserpine occur equally in the three textbook series. In addition, five topics occur together in Aktories and Lüllmann, and two topics occur together in Aktories and Karow. These topics do not occur in the other textbook series. With 12 topics, almost half of the total of 26 topics concerning reserpine are reserved for only one textbook series. These topics do not appear in the other two textbook series (topics only in Lüllmann, 4; topics only in Aktories, 5; topics only in Karow, 3). If one takes the editions of a textbook series together, with 17 different topics, more topics are addressed in Aktories than in Lüllmann (16 topics) and Karow (13 topics).

**Fig. 6:**
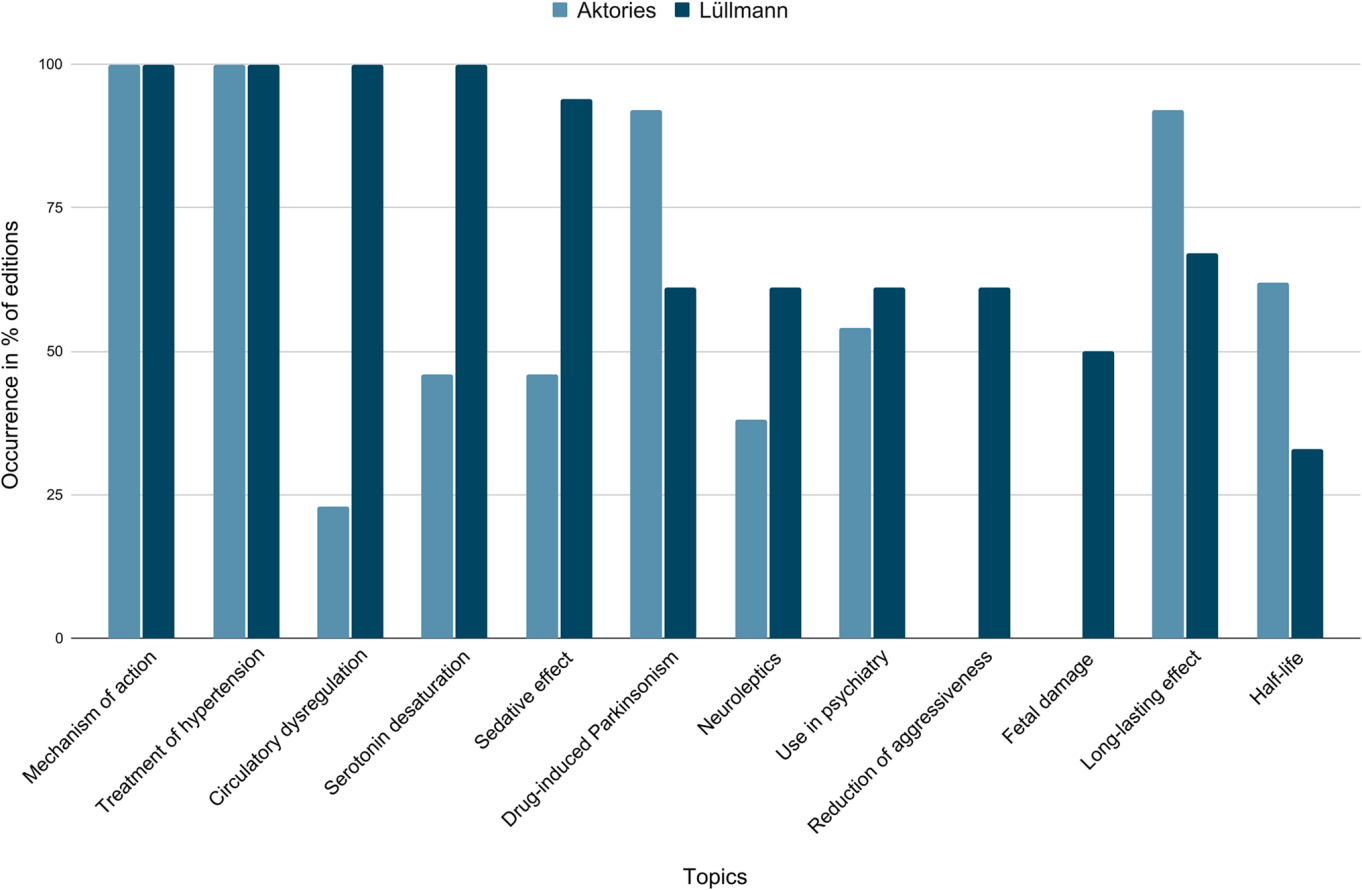
Most frequent topics: the chart shows the most frequent topics in context with reserpine and in how many percent of the editions they occur. A common topic appears in at least one textbook series (Aktories or Lüllmann) in at least 50% of the editions

In addition, it was investigated which are the most frequent topics in the textbooks (in at least 50% of the editions of Aktories or Lüllmann). Figure 6 shows that the most common topics are the mechanism of action and the treatment of hypertension. They appear in all editions of the textbooks. Additionally, there are other topics that are addressed in many editions. These are, for example, circulatory dysregulation and serotonin desaturation (100% of the editions in Lüllmann). Other frequently mentioned topics regarding reserpine are the sedative effect, drug-induced Parkinson’s syndrome, neuroleptics, use in psychiatry, reduction of aggressiveness, harm to the foetus and embryo, the long-lasting effect and the half-life.

In summary, reserpine is particularly present in the chapter group “nervous system/antisympathotonics”. In addition, most entries can be described with the keyword mechanism of action. However, with 26 different topics, considerably more aspects are addressed in the textbooks. Furthermore, some topics can be identified that appear particularly frequently in the textbooks.

### Presentation of reserpine

Textbooks also use diagrams, tables and structural formulae for didactic support, here referred as graphical presentations. Figure S4 shows how Lüllmann uses tables, diagrams and structural formulae in its editions. The number of graphical presentations in Lüllmann also decreased after a peak in editions 9 and 10. Up to edition 10, one structural formula is always presented per edition; after that, no more. The number of diagrams increases, especially up to edition 13, with three diagrams. Tables are also used in some editions.

Figure S5 shows the development of the ratio of graphical presentations per number of entries in the three textbooks. In Lüllmann, there is a slight increase from 0.2 in 1964 to 0.33 in the current edition of 2016, with a peak in 1999 of 0.8. In Aktories, the ratio of graphical presentations per number of entries decreases over time. Starting from 1.2 in 1975, the ratio decreases to 0.375 in 2022. In Karow, the ratio remains at 1 across all editions with no changes. Therefore, it uses more graphical presentations per entries than the other textbooks, which further underlines the characteristic of Karow as a reference book.

Figure S6 shows the proportion of diagrams among the graphical presentations in the Lüllmann and Aktories. Karow does not use diagrams in any of its editions. In Lüllmann and Aktories, an increase in the proportion of diagrams can be observed up to the 13th edition of both textbooks. This may be explained by the demand for a modern didactic approach to textbooks.

### ADRs of reserpine

**Fig. 7:**
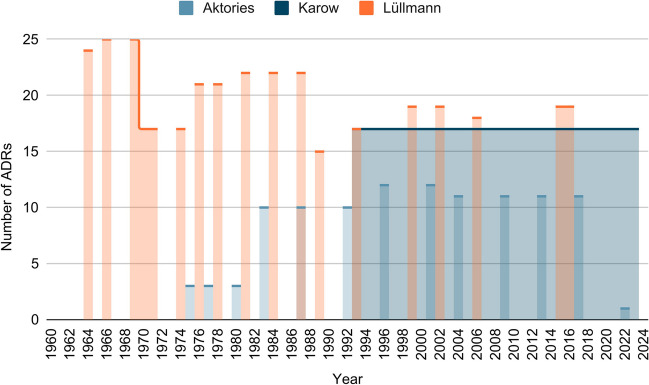
Number of mentioned ADRs: the graph shows the number of ADRs regarding reserpine mentioned per edition of the textbooks (Lüllmann, Aktories, Karow)

Figure 7 shows the development of the number of ADRs mentioned in the three textbooks. In Lüllmann, the number of ADRs mentioned decreases slightly over time, but more ADRs are mentioned than in the other two textbooks. For example, 25 ADRs are mentioned in 1966 and 1970 and in 2016, only 19 ADRs. The number of ADRs mentioned in Karow remains constant at 22. In Aktories, an increase in the number of ADRs mentioned can be observed from three ADRs in 1975 to 12 ADRs in 2001, as well as a sharp drop from the penultimate edition (11 ADRs in 2017) to the current edition (1 ADR in 2022). However, Aktories mentions fewer ADRs than Karow or Lüllmann.

**Fig. 8:**
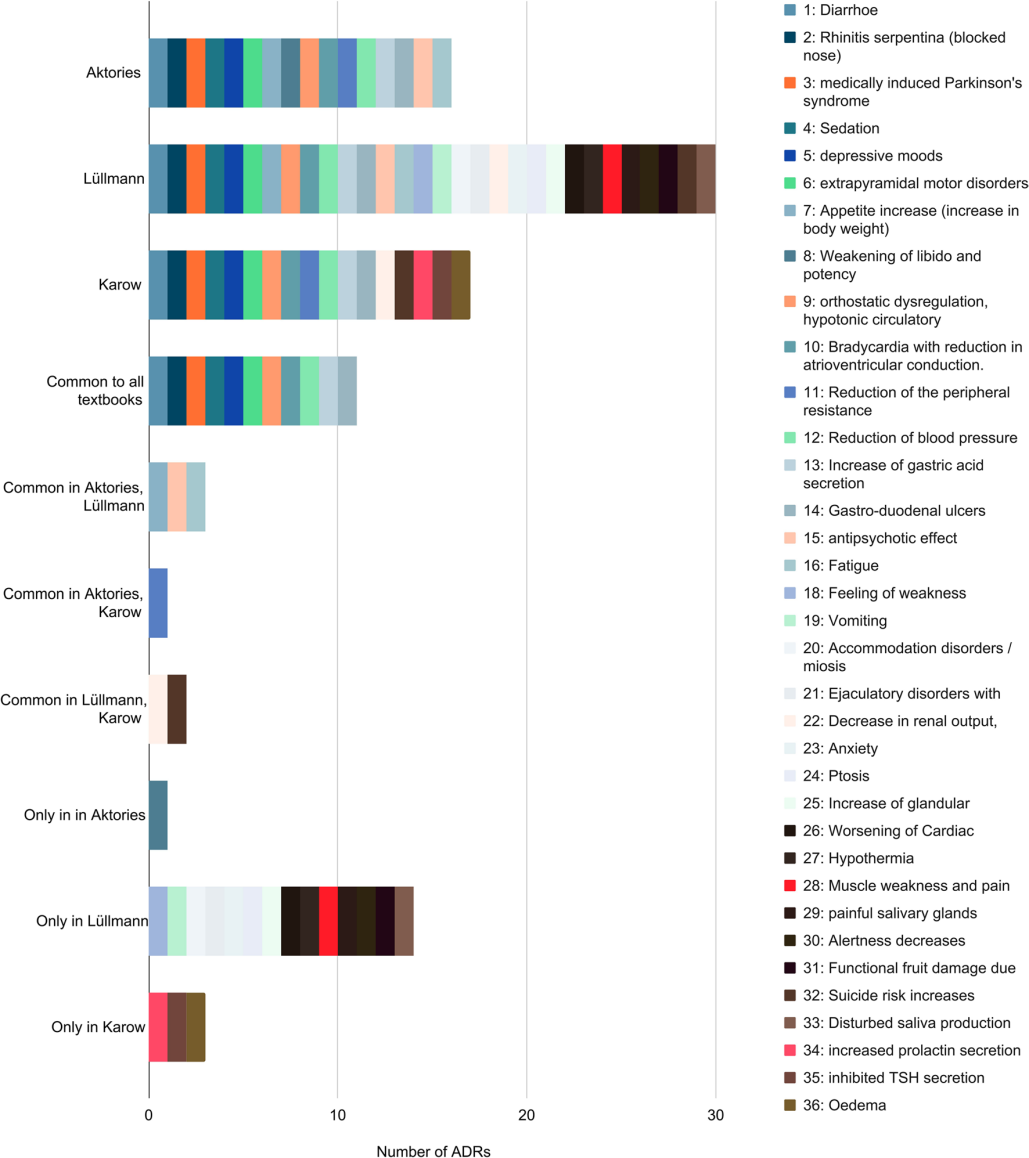
ADR in textbook comparison: the chart shows in which textbooks (Lüllmann, Aktories, Karow) the mentioned ADRs regarding reserpine occur

Figure 8 shows which ADRs occur in which textbook series and which of these ADRs are also common to certain textbook series. Adverse reactions that are mentioned in all textbook series are, for example, diarrhoea, rhinitis serpentina, drug-induced Parkinson’s syndrome, sedation and depressive moods. Other ADRs are reserved for individual textbook series. Especially in Lüllmann, with 14 ADRs, some are mentioned that do not appear in the other textbooks. The number of ADRs mentioned exclusively in Aktories (1) and Karow (3) is significantly lower.

### Changes compared to previous editions

Figure S7 (Aktories), Fig. S8 (Lüllmann) and Fig. S9 (Karow) show the changes to previous editions in the three textbook series by means of coding. In Lüllmann and Aktories, there are some changes between the editions. For example, in Aktories in the 7th edition, with nine new entries, a particularly large number of changes compared to the previous edition can be observed (Fig. S7). In Lüllmann, there are three new entries and two extensively revised entries in the 7th edition compared to the previous edition, which is also a particularly large number of changes (Fig. S8). In Lüllmann and Aktories, no edition is completely identical to the previous edition regarding reserpine. This is shown by the fact that some of the entries are completely new, while others have been extensively revised and by the occurrence of only minor changes in wording. The situation is different in Karow (Fig. S9). Here, apart from minor changes in wording with one new entry (edition 18), only one major change occurs. Thus, many editions regarding reserpine in the Karow are completely identical to their previous edition.

### Dosages of reserpine

An application of reserpine without a combination partner is described in Lüllmann until 1976 (7th edition) in a daily dosage of 0.25–1 mg (Appendix 3). A dosage in the context of a combination therapy is also described in Lüllmann until 1976 (7th edition) in a dosage of 0.3 mg/day. Dihydralazine is given as a combination partner in a dosage of 30 mg/day (Appendix 3). In Aktories, a daily dosage of 0.1–0.3 mg is described until 1987 (5th edition) with a diuretic for basic therapy (Appendix 2). Even in the current edition of Karow (2023, edition 31), the dosage of 0.05/0.1 mg reserpine in the drug Briserin with clopamide (2.5/5 mg) as a combination partner is still given. Here, it is also mentioned that other preparations sometimes use other combination partners (hydrochlorothiazide or chlortalidone, sometimes also dihydralazine). The dosage of reserpine in Briserin is still mentioned in 2017 in Aktories (12th edition) (Appendix 1). Until 1989, the daily dose of reserpine was given as 0.1–0.3 mg in Lüllmann (12th edition) (Appendix 3).

An impairment of occupational performance, even at doses of 0.1–0.3 mg, is described in Lüllmann until 1987 (11th edition). Due to the risk of developing depression, a daily dose of 0.25 mg should only be exceeded for a short time (1981, 9th edition Lüllmann) (Appendix 3).

A different view can be observed in Aktories. In 2017 (12th edition), it is still described that the ADRs up to 0.25 mg/day are low. Adverse effects were observed, according to the edition, especially at daily doses of 0.75 mg in the 1960s and 1970s. It is also mentioned that reserpine was formerly used as a neuroleptic in doses of up to 5 mg/day (Appendix 2).

In summary, an application of reserpine is described in the textbooks almost exclusively in a combination therapy. The greatest discrepancy regarding the dosage in the textbooks can be seen between Lüllmann and Aktories. In 1987, for example, Lüllmann described an impairment of occupational performance already at doses of 0.1–0.3 mg/day. Aktories, on the other hand, states in 2017 that there are only slight ADRs at doses of 0.25 mg/day.

### Ttrade names of drugs containing reserpine

Table 2 shows trade names which are mentioned in the individual editions of the three textbook series. With eight trade names, Lüllmann mentions the most. In contrast, only Briserin is mentioned as a trade name in Karow. Aktories mentions three trade names. It is striking that no trade names are mentioned in Lüllmann in the 16th–18th edition (i.e. up to the current edition) or in Aktories in the current edition. In contrast, in Karow up to the current editionthe trade name Briserin is mentioned. A discrepancy can be seen between Briserin and Briserin N. For example, only in Aktories Briserin N is mentioned, but not in the other two textbooks. However, as the prescription figures show, Briserin N is the drug that has been prescribed the most in recent decades. In addition, Briserin has not been used for a long time, so it could be that actually Briserin N is meant in the textbooks.

**Table 2:** Trade names of reserpine-containing drugs in the textbooks. The table shows in which editions of the textbooks the different trade names are mentioned

Trade name	Karow	Lüllmann	Aktories
Briserin	12–13th edition	14–15th edition	-
Briserin N	-	-	8–12th edition
Sedaraupin	-	1–10th edition	1–5th edition
Serpasil	-	1–12th edition	4–5th edition
Modenol	-	14–15th edition	-
Tri-Thiazid-Reserpin	-	14–15th edition	-
Darebon	-	14–15th edition	-
Disalpin	-	14–15th edition	-
Durotan	-	14–15th edition	-

In addition, the trade names are linguistically interesting. Appendix 4 shows a linguistic analysis of the trade names and illustrates how suggestive they are. For example, the name Sedaraupin (lat. sedare) indicates a sedative effect and Modenol (lat. modererre) an attenuating effect. The ending of briserine is strongly reminiscent of the ending of reserpine, and the prefix of serpasil could refer to snake root (*Rauwolfia serpentina*).

### Indications of reserpine

**Fig. 9:**
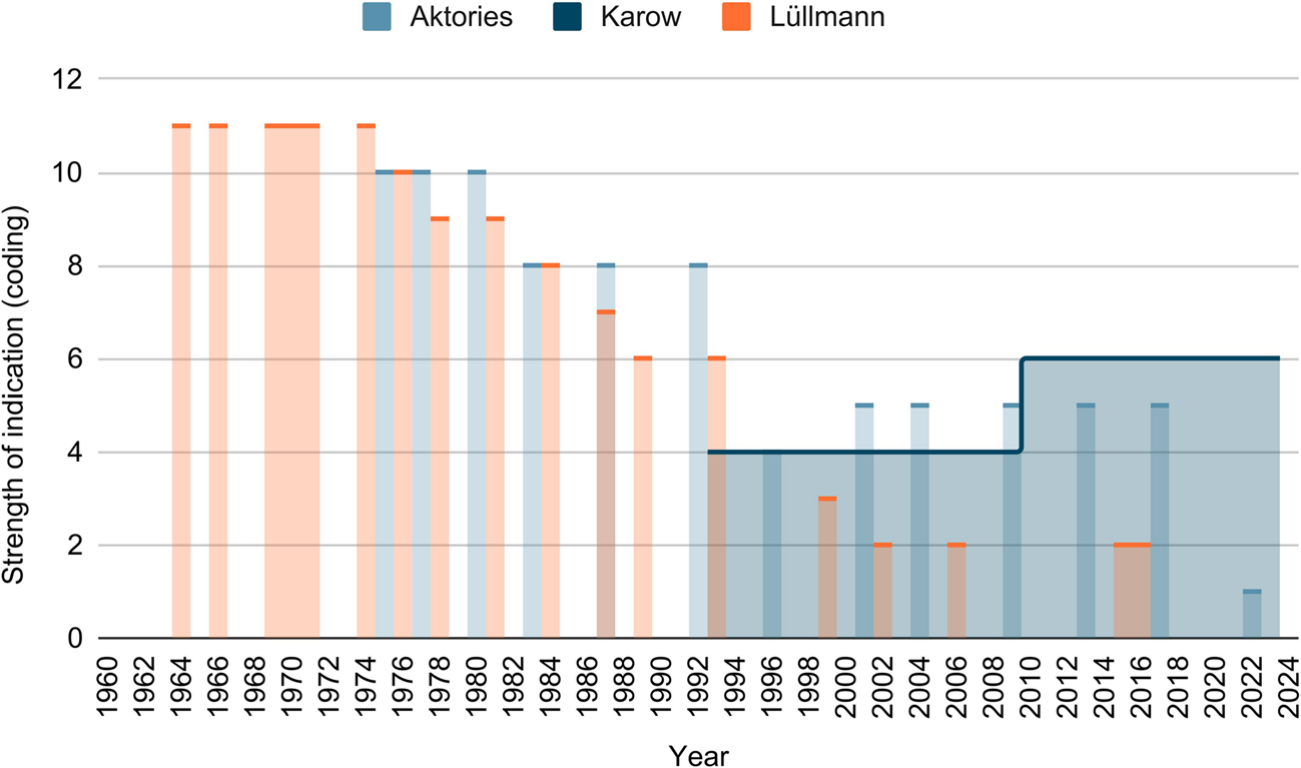
Strength of indication (hypertension treatment): the graph shows the strength of indication (treatment of hypertension with reserpine) of the different editions of the three textbooks (Lüllmann, Aktories, Karow) according to the coding explained under methods

Figure 9 shows the development of strength of indications for the treatment of hypertension in the three textbook series. A high coding expresses a greater strength of recommendation and a lower coding a more restricted indication up to obsolescence. Thus, one can see how the strength of indication in Aktories and Lüllmann decreases over time. The indication therefore becomes more and more restricted. A code of 3 or lower indicates that reserpine is considered obsolete. Therefore, in Lüllmann, reserpine is described as obsolete in 1999 and in Aktories, only in the latest edition of 2022. In Aktories, reserpine is still not described clearly as obsolete in 2017. In Karow, one indication is still mentioned in each edition. The indication mentioned is limited to therapy-refractory hypertension.

Only in Lüllmann the indication of reserpine for the treatment of hypertension is not restricted until 1974. From 1976 to 1999, the indication in Lüllmann is further restricted. First of all, the side effects are brought more into focus, and then the use is increasingly restricted to therapy-refractory forms of hypertension with increasing severity. This development can also be applied to the editions of Aktories from 1975 to 2017. Overall, however, it can be stated that in Aktories, the indication is not restricted as much as in Lüllmann during this period.

**Fig. 10:**
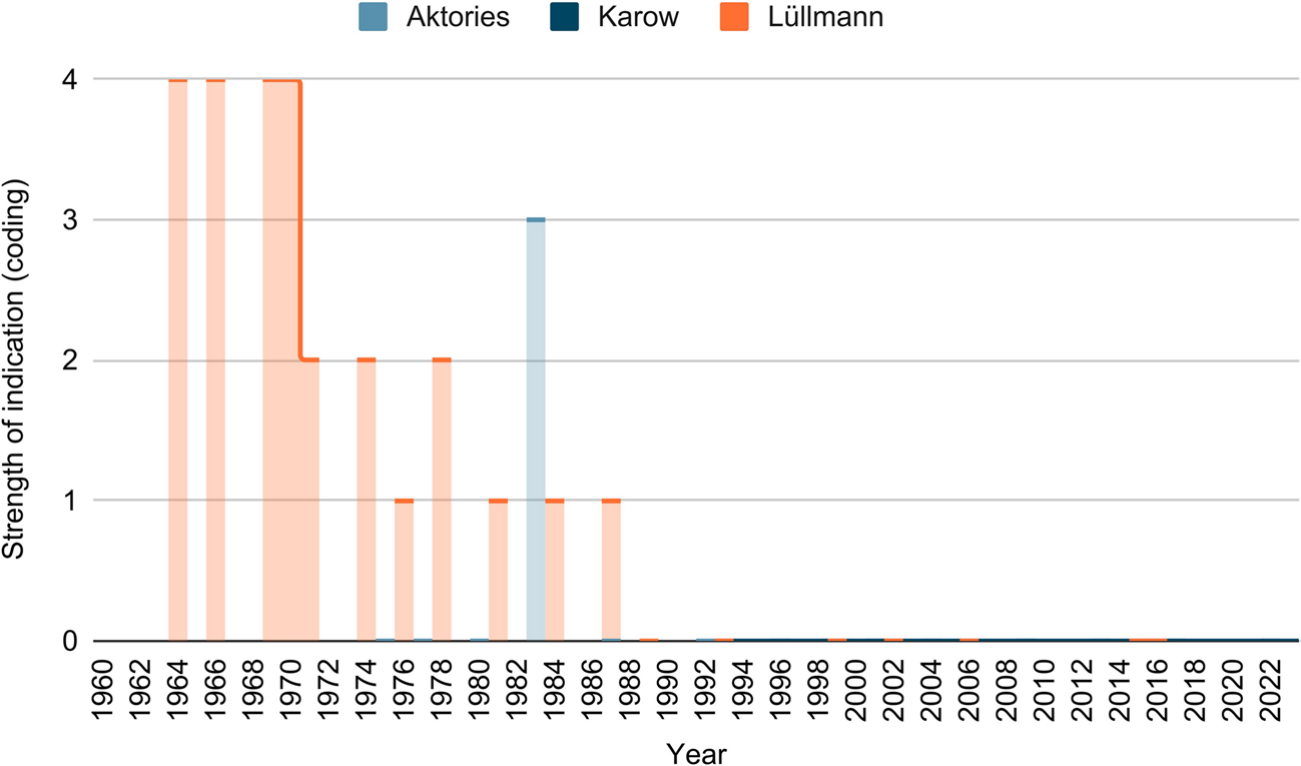
Strength of indication (treatment of psychoses): the graph shows the strength of indication (treatment of psychoses with reserpine) of the different editions of the three textbooks (Lüllmann, Aktories, Karow) according to the coding explained under methods

Figure 10 shows the development of the strength of indications for the treatment of psychoses in the three textbook series. Here, too, a high coding refers to a greater strength of recommendation. Only Lüllmann mentions an indication for reserpine in the treatment of psychoses until 1970. However, even here, other drugs are preferred for treatment. In 1983, Aktories describes that reserpine was used earlier in psychiatry. In Karow, nothing is mentioned about the former indication.

Thus, the indication for the treatment of psychoses has been abandoned in the textbooks for a long time. In contrast, the textbooks differ in the indication for the treatment of hypertension, whereby it has been more restricted over time (exception in Karow).

### Conceptual differences between the textbooks

The textbook Karow currently has 31 editions, which are published annually. The first edition appeared in 1993 (Karow and Lang 1993). Lüllmann and Aktories have longer and more irregular publication intervals. Lüllmann is the oldest of the three textbooks, its first edition was published in 1964 (Kuschinsky and Lüllmann 1964). The current edition (18th edition) is from 2016 (Lüllmann et al. 2016). The first edition of Aktories was published 11 years later (Forth et al. 1975), and the current edition (Aktories et al. 2022) is from 2022.

Aktories and Lüllmann are similar in their presentation. Reserpine is mentioned in different chapters, and the texts are supported by various diagrams. In comparison, the information on reserpine in Karow is presented much more concentrated. There is also a big difference in the presentation compared to the other two textbooks. For example, in Karow, almost exclusively key points are used, whereas in Lüllmann and Aktories, the information is formulated in a text. Karow has more characteristics of a reference book, in which the information is summarised briefly and concisely and is easier to see at a glance.

In Aktories and Lüllmann, new entries, heavily revised or even only minor changes in wording occur in the different editions. This contrasts with Karow. Here, there are only minor differences between the different editions. Of course, this is also due to the publication interval of only 1 year for Karow. Despite this, hardly any differences can be observed between the 1st and 31st editions, although there are 30 years between them.

In Karow, there are only three entries in the index on reserpine in the current edition (2023). In Lüllmann and Aktories, there are significantly more entries in the keyword index on reserpine. The maximum is 12 entries in Aktories (in 1996) and 8 entries in Lüllmann (1981 and 1984). Karow does not differ so much from the other textbooks in the length of text per edition concerning reserpine. Thus, Karow is just over one page and the Lüllmann in its last five editions around 0.75 pages. In comparison, the last five editions of Aktories have a text length of over 2 pages in some cases (whereby one must exclude the last edition). The text length per edition (concerning reserpine) develops differently over time in Lüllmann and Aktories. Thus, apart from the last edition, an increase in text length can be observed in Aktories, whereas a reduction in text length can be observed in Lüllmann. In the earlier editions of Lüllmann, the text length was almost 2.5 pages (1978), whereas in the current edition (2016), the text length is less than 0.5 pages. The text length concerning reserpine is less than half a page in the 2nd edition of Aktories (1977), whereas in the penultimate edition (2017), it is over 2 pages.

In all three textbooks, most of the chapters addressing reserpine can be grouped under “nervous system/antisympathotonics”. In Aktories, the entries in this chapter group increase over time, in contrast to Lüllmann. In Karow, the number of different chapters is significantly lower than in Lüllmann and Aktories. Even though there are significantly fewer entries in Karow concerning reserpine, only slightly fewer topics are addressed than in the other two textbooks. Thus, the information on reserpine is more concentrated in Karow (Aktories 17 topics, Lüllmann 16 topics, Karow 13 topics).

The three textbooks contain different forms of “graphical presentations” (structural formula, diagrams, tables). In Lüllmann and Aktories, there are diagrams, tables and also the structural formula of reserpine is shown. In Karow, on the other hand, only tables relating to reserpine are shown, which supports the character of a reference work. In Aktories and Lüllmann, the proportion of diagrams among the graphic presentations increases in comparison from more recent to older editions. This may be explained by the demand for a modern didactic presentation.

In Lüllmann, more ADRs are mentioned than in the other two textbooks. Some ADRs are also common to all three textbooks. These are, for example, rhinitis serpentina, diarrhoea, depression or the drug-induced Parkinson’s syndrome.

With regard to the dosage information on reserpine, the discrepancy between Aktories and Lüllmann is particularly interesting. Thus, in 1987, Lüllmann describes an impairment of occupational performance already at doses of 0.1–0.1 mg/day. Aktories, on the other hand, claims in 2017 that ADRs are only slight at doses of 0.25 mg/day.

That reserpine is indicated for the treatment of psychoses is only described in Lüllmann until 1970. In Lüllmann and Aktories, the indication for the treatment of hypertension is increasingly restricted over time, whereby this restriction, up to obsolescence, is even more pronounced in Lüllmann. In both textbooks, however, reserpine is marked as obsolete in the respective current edition. Only in Karow is the indication for therapy-refractory forms of hypertension still mentioned for reserpine in the current edition.

### Representation of reserpine in English textbooks

In order to illustrate how reserpine is presented in English textbooks, three textbooks were cursorily analysed in relation to reserpine. The textbooks are *Rang & Dale’s Pharmacology* (Ritter et al. 2019), *Basic & Clinical Pharmacology* (Katzung 2017) and *Goodman & Gilman’s The Pharmacological Basis of Therapeutics* (Brunton and Knollmann 2022).

In *Rang & Dale’s Pharmacology* (Ritter et al. 2019), it is described that reserpine blocks the vesicular norepinephrine transport and reduces the dopamine concentration in the brain. Nowadays, reserpine is only used experimentally, but it was used to treat hypertension in former times. In higher doses, reserpine causes depression. Reserpine is now considered obsolete because of its ADRs. In addition, reserpine’s role in the development of the monoamine hypothesis in the development of depression is explained. In the course of this, the effectiveness of reserpine in reducing symptoms in schizophrenia and how it can prevent amphetamine-induced behaviour change are outlined.

In *Basic & Clinical Pharmacology* (Katzung 2017), the mechanism of action, the reduction of noradrenaline concentration through inhibition of vesicular transport as well as the reduction of serotonin is also presented. The use of reserpine in the treatment of hypertension is described, and side effects such as sedation, diarrhoea, medical-induced Parkinson syndrome and depression are listed. Although the use is described as rare, a designation of reserpine as obsolete is not made. In addition, pharmacokinetic data on reserpine are given, and a use in the treatment of Huntington’s disease is described.

In Goodman & Gillman’s (Brunton and Knollmann 2022), the mechanism of action in the reduction of monoamines such as noradrenaline and serotonin is described. In particular, inhibition of the vesicular transporter VMAT2 is mentioned. It is described that reserpine was formerly used for the treatment of hypertension and psychosis, but it is no longer used today. The history of reserpine and its long-lasting effect are explained. Among the ADRs, depression is emphasised. Depression is rare at doses of 0.25 mg/day, but ADR has been observed even at such doses. Reserpine has a good effect in elderly patients with systolic blood pressure but has been replaced by superior drugs and is no longer recommended for the treatment of hypertension.

Compared to Aktories, Lüllmann and Karow, the presentation and topics mentioned are very similar. However, topics are also mentioned that do not appear in the German textbooks, such as the application in the therapy of Huntington’s disease.

###  Prescription data of reserpine

**Fig. 11:**
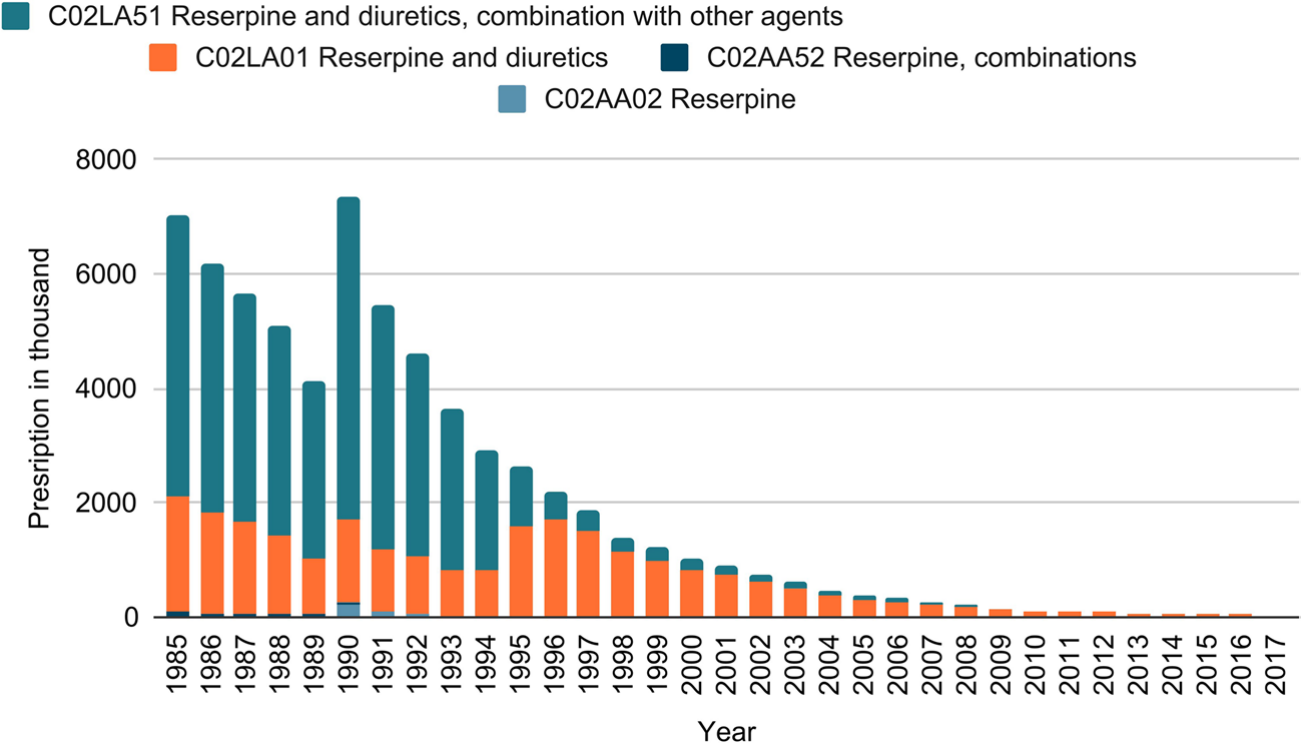
Prescription numbers: the graph shows the number of prescriptions (in thousands prescriptions) of drugs containing reserpine as the active agent per year. The data are broken down by ATC code. The data were provided by the WIdO and contain information regarding prescriptions of reserpine from 1985 to 2017 in Germany

Figure 11 shows the development of the prescription figures for drugs containing reserpine from 1985 to 2017, broken down by ATC code. The ATC code divides drugs into different groups with regard to therapeutic aspects. Prescription numbers of reserpinedecreasedover time since 1990. By 2016, the number of prescriptions had fallen to 70.5 thousand prescriptions. Until 1994, the majority of the prescription figures were accounted for by prescriptions for ATC code C02LA51 (reserpine and diuretics, combination with other agents). From then on, the majority of prescriptions were for ATC code C02LA01 (reserpine and diuretics). From 2008 onwards, there were no prescriptions for other ATC codes. The share of prescriptions for the ATC codes C02AA52 (reserpine, combinations) and C02AA02 (reserpine) in the total prescriptions is only marginal.

The prescription figures from 1990 onwards can be approximately described with an E-function (Fig. 1).

Figure S10 shows the number of different preparations containing reserpine per year and broken down by ATC code. Since 1985, more than 20 different drugs (standard aggregate names) have been prescribed per year in the following years. Here, too, with the number of prescriptions, a peak can be observed in 1990 with 24 different drugs. By 2017, the number of prescribed drugs had decreased to one. In addition to the renewed increase in 1990, an increase in 2001 is also noticeable. Most drugs are distributed approximately equally between the ATC codes C02LA51 (reserpine and diuretics, combination with other agents) and C02LA01 (reserpine and diuretics). The number of drugs for ATC code C02AA02 (reserpine) per year is lower than for the two previously mentioned ATC codes. Only one drug is prescribed annually up to 1995 for ATC code C02AA52 (reserpine, combinations).

It is also interesting to observe which drugs were used over a period of time. The prescription figures, received from the WIdO, show that some drugs such as Briserin N, Bendigon N or also Adelphan-Esidrix were prescribed over a very long period of time. Other drugs, such as Repicin or Rausedan, were only prescribed for a few years.

**Fig. 12:**
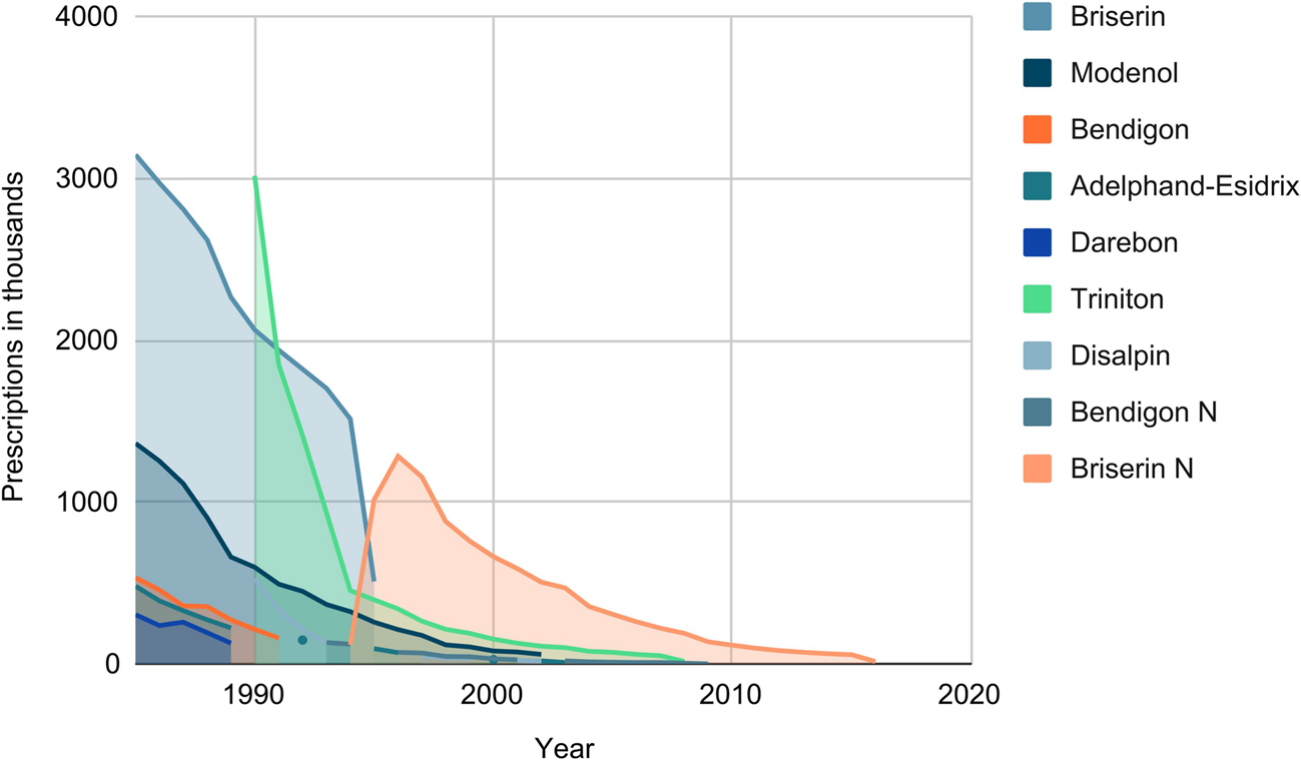
The 5 most prescribed standard aggregate names per year: the graph shows the prescription numbers (in thousands prescriptions) of the five drugs that had the highest prescription numbers containing reserpine as the active agent per year. The data is provided by the WIdO and contains information regarding prescriptions of reserpine from 1985 to 2017 in Germany

Figure 12 shows the five drugs with the highest number of prescriptions per year. Briserin was particularly dominant in the number of prescriptions from 1985 to 1995. The maximum in the recorded data was 3145.6 thousand prescriptions in 1985. Triniton was listed for the first time in 1990 and was even stronger in terms of prescriptions than Briserin. Brisein N had a very strong market dominance since the mid-1990s and was the only reserpine-containing drug prescribed from 2010 to 2017. The maximum number of prescriptions for Briserin N was 1282.4 thousand in 1996. Modenol, Bendigon N, Disalpin, Darebon, Adelphan-Esidrix and Bendigon also had a large market presence in terms of number of prescriptions.

**Fig. 13:**
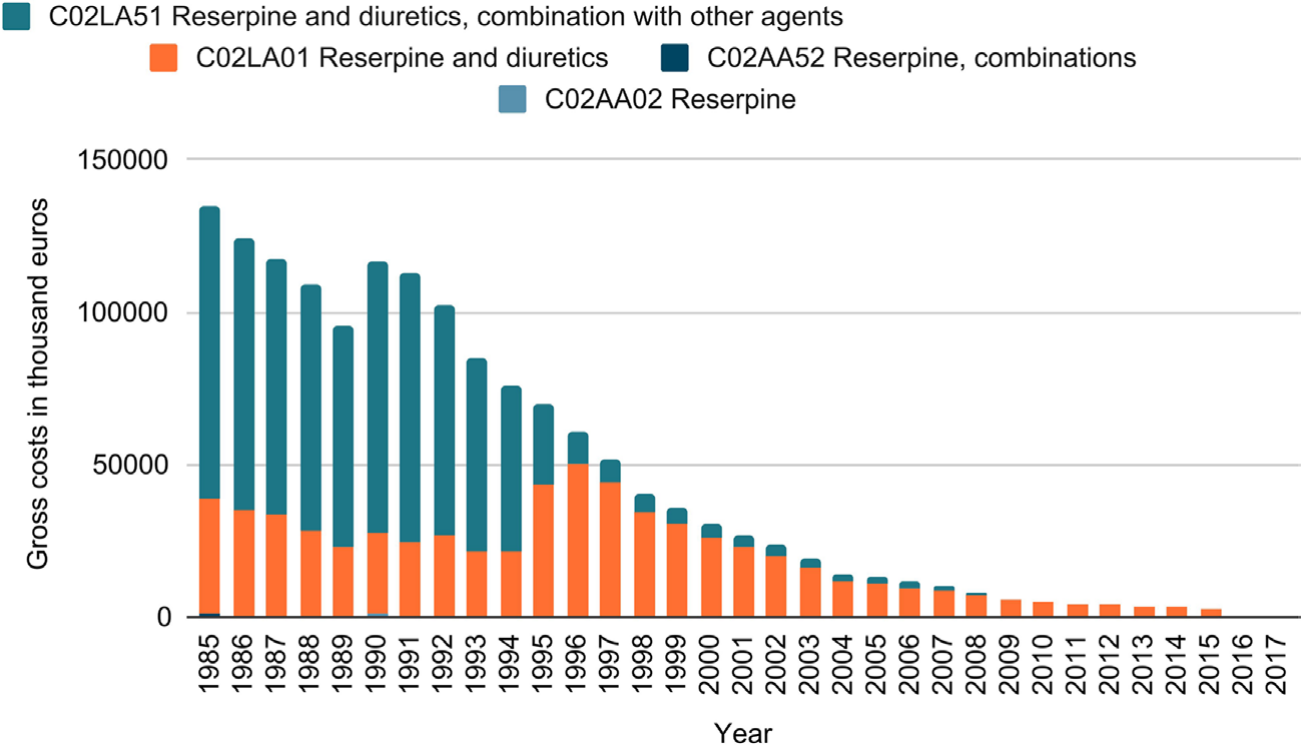
Gross costs per ATC code: the graph shows the gross costs (in thousands of euros) of drugs containing reserpine as an active agent per year. The data is provided by the WIdO and contains information regarding prescriptions of reserpine from 1985 to 2017 in Germany

Figure 13 shows the development of the gross costs of reserpine-containing drugs, broken down by ATC code. A similar picture emerges here. In 1985, the gross costs in thousands of euros amounted to 134,949.50 and fell to 0.2 by 2017. Here, too, an increase can be observed in 1990, which, however, is lower than in the prescription figures per year. In addition, a similar distribution among the different ATC codes can be seen. Until 1994, the largest gross costs were accounted for by the ATC code C02LA51 and in the following years, almost exclusively by the ATC code C02LA01. The gross costs attributable to ATC codes C02AA52 and C02AA02 are again only marginal.

Figure S11 shows the gross costs in euros per daily dose (DDD) of all prescribed drugs containing reserpine, broken down by ATC code. In total, 28 different drugs containing reserpine were prescribed from 2010 to 2017. The average gross cost per DDD is €0.28. Most of the drugs are listed under the ATC codes C02LA51 and C02LA01. Only one drug was prescribed to ATC code C02AA52 and three to ATC code C02AA02.

Compared to the trade names mentioned in the textbooks, it is noticeable that there are significantly more drugs on the market than are mentioned in the textbooks. Karow is standing out here, where only Briserin is mentioned as a drug (even in the current edition, although it has not been on the market for 6 years), and even Aktories mentions in all its editions only three drugs with reserpine. The distinction between Briserin and Briserin N seems to be inaccurate in the textbooks. Thus, in Karow and Lüllmann, the more recent editions mention Briserin N instead of Briserin. Only Aktories lists Briserin N (Table 2).

In summary, as the prescription figures show, reserpine was widely used in the past. However, it became less and less important towards the end of the 1990s and has not been prescribed at all since 2017.

###  Hypertension guidelines concerning reserpine

In order to check the textbook recommendations for timeliness, they were compared with guidelines on hypertension treatment. No mention of reserpine is made in the following guidelines: “Guideline for the pharmacological treatment of hypertension in adults” of the World Health Organization (WHO) (2021); “Global Hypertension Practice Guidelines” of the International Society of Hypertension (2020); “Pocket Guidelines Management der arteriellen Hypertonie” of Deutsche Hochdruckliga, Deutsche Gesellschaft für Kardiologie, European Society of Cardiology and European Society of Hypertension (2014 and 2018) and “Arterielle Hypertonie” of Deutsche Hypertonie Gesellschaft/Deutsche Liga zur Bekämpfung des hohen Blutdrucks (1998).

In “Arterial Hypertension” of the World Health Organization (WHO) (1978), the use of reserpine is mentioned as part of a step therapy. In the second stage, treatment is given in combination with a diuretic or beta-blocker. In the third stage, treatment is in combination with a diuretic and hydralazine. The mechanism of action of reserpine is also explained in the guideline. It consists of a central and peripheral component, whereby the peripheral component is responsible for the blood pressure-lowering effect. Adverse effects include sedation, depression, dry mouth, nasal congestion and other adverse effects. A reduction in potency and libido has also been described.

In “Leitlinien für die Prävention, Erkennung, Diagnostik und Therapie der arteriellen Hypertonie” of Deutsche Liga zur Bekämpfung des hohen Blutdruckes e.V. (Deutsche Hochdruckliga) (2003), reserpine is mentioned in the context of a combination therapy with a diuretic. The guideline also compares the costs of different preparations. Regarding reserpine, it states that no monopreparations are offered in Germany.

“Arterielle Hypertonie” 2nd edition of Arzneimittelkommission der deutschen Ärzteschaft (2004) reported that reserpine has largely lost its importance despite its still widespread use. If patients have been satisfactorily adjusted with reserpine for a long time, treatment with reserpine can be continued. The ADRs are described in more detail. In addition, it is said to be unsuitable in pregnancy.

As in the 7th edition (1976) of Lüllmann, the combination of reserpine with dihydralazine is also described in the WHO guideline of 1978. In Aktories, as well as in the WHO guideline (1978), the combination with a diuretic in the case of a contraindication to beta-receptor blockers (β-adrenoreceptor antagonists) is mentioned until the 5th edition (1987). As in the guidelines, reserpine is not a first-line agent for the treatment of hypertension. Reserpine is no longer mentioned in the current guidelines, and in 2004, the guidelines of Arzneimittelkomission der deutschen Ärzteschaft also describes that reserpine has largely lost its importance. In Aktories, reserpine is only described as obsolete in the current edition, but not yet in 2017. Even in the current edition of 2023, Karow does not label reserpine as obsolete. Thus, an indication for reserpine has been mentioned in the textbooks for much longer than the guidelines indicate. This is particularly striking in Karow. Lüllmann should be distinguished from this. Here, it is described that reserpine is obsolete as early as 1999 and is thus closer to the indication of the guidelines.

The ADRs mentioned in the guidelines can also be found in the textbooks.

In summary, it can be stated that the textbooks still give an indication (such as in the current edition of Karow) for reserpine, although there are no drugs containing reserpine left on the German market and reserpine actually no longer has any relevance according to the guidelines. Lüllmann should be distinguished from the other two textbooks, as it shows significantly fewer discrepancies with the guidelines than Aktories and especially Karow.

###  Analysis of IMPP questions, Viamedici and Amboss

Analysis of prescription figures and hypertension guidelines shows that reserpine is no longer relevant in today’s therapy. Nevertheless, reserpine is still treated in textbooks. The question arises what the reasons for this might be. One obvious assumption is that reserpine is considered relevant for examinations and is still asked for. This hypothesis was tested with the analysis of IMPP questions. The Institute for Medical and Pharmaceutical Examination Questions (IMPP) is responsible for the design of examination questions in the written part of the state examinations in the study of medicine in Germany. The IMPP questions were used from the online learning programme Viamedici.

Viamedici is an online learning programme of the Thieme Group and contains the official IMPP questions. Our search did not yield any questions that dealt with reserpine. It seems that reserpine is not considered relevant for the examination by the IMPP and so it is also not mentioned in the IMPP list of drugs (https://www.impp.de/informationen/presse/id-2022/id-2023/arzneistoffliste.html, last accessed 11 October 2023).

The reasons why reserpine still appears in textbooks must therefore be different. Possible reasons include the fact that reserpine is well suited as an example for understanding the mechanism of action of antisympathotonics. Even though it is no longer used today, interactions with other drugs and diseases are still important. Moreover, it is of historical interest. In former times, reserpine was widely used in therapy and was important for basic research into depression and Parkinson’s disease. Until now, reserpine is still used in research.

Reserpine is mentioned in different fields in Viamedici:

- Antisympathotonics.
- Biogenic amines (serotonin).
- Possible drug cause of loss of libido.
- Simultaneous administration of L-dopa and reserpine in Parkinson’s treatment is contraindicated.
- Drug-induced lupus erythematosus.
- Drug-induced hyperprolactinaemia.
- Reduction of insulin requirement in type 1 diabetes mellitus.
- Drug-induced priapism.

Reserpine is treated particularly in this context among the antisympathotonics. The mechanism of action of reserpine is explained, and adverse effects and past indications are also mentioned. As a current indication, therapeutic use in fixed combination with a diuretic (e.g. the thiazide diuretic clopamide) is mentioned. Otherwise, the therapeutic application of reserpine is obsolete today. Nevertheless, an indication for hypertension treatment is still mentioned in Viamedici. The Viamedici learning programme seems very similar to the textbooks in terms of topics and content as well as in the preparation of reserpine in the individual chapters.

In the online learning programme Amboss, reserpine is only mentioned in a table on antisympathotonics. The mechanism of action and ADRs (orthostatic dysregulation, depression, gastrointestinal ADRs) is explained here. A speech bubble on reserpine emphasises that it is not routinely used and is only used as part of combination therapy treating hypertension.

Compared to Viamedici, on Amboss, reserpine is presented in a similar but reduced way.

### Relevance of reserpine in research

To show how reserpine is developing in research, the mentions of reserpine in PubMed were analysed. PubMed is an online database for medical and biomedical content.

A decrease in the number of mentions of reserpine was observed (Fig. 1). Despite the fact that the relevance of reserpine in research has declined, it is still mentioned . As an example, three different reviews were selected and analysed to illustrate the relevance of reserpine for research today.

In 2016, a study compares reserpine and chlorpromazine for the treatment of schizophrenia (Nur and Adams 201610.1002/14651858.CD012122.pub2, last accessed 11 October 2023). According to the authors, chlorpromazine is superior to reserpine. By today’s standards, the conclusions of past studies comparing the two drugs are of limited quality. Moreover, in this study, reserpine is described as almost obsolete nowadays.

According to another study from 2016, reserpine can lower well systolic blood pressure. No clear conclusions could be drawn on the dose-response pattern due to a small number of randomised controlled trials (Shamon and Perez 201610.1002/14651858.CD007655.pub3, last accessed 11 October 2023).

In addition, a study from 2022 was analysed (Strawbridge and Javed 202310.1177/02698811221115762, last accessed 11 October 2023). According to this study, reserpine is considered obsolete, especially due to causing depression. The assumption that reserpine causes depression was the basis of the monoamine hypothesis in the development of depression. However, according to the study, the links between depression and reserpine use are inconsistent and lack clear evidence. The authors conclude that the monoamine hypothesis is too simplistic in explaining the triggering of depression and needs to be revised.

## Limitations

Due to the conception of a textbook, the key index was used as the basis of the analysis. In consequence, one must rely on the fact that all contents relating to the drug are listed in the index. Any content that was not listed in the index was not taken up. In addition, editions five and eight of Karow could not be analysed due to procurement problems. However, since the pages concerning reserpine in editions four and six as well as seven and nine are identical and the differences between the editions in Karow are only marginal, this should not have a substantial influence on the results. A comparison with guidelines has also proved problematic in some cases. After enquiries at the Arzneimittelkommission der deutschen Ärzteschaft AkdÄ, the Deutsche Hochdruckliga e.V. and the Arbeitsgemeinschaft der Wissenschaftlichen Medizinischen Fachgesellschaften e.V., these institutions could only provide a selection of guidelines. Additionally, many guidelines were also selected through an internet search. Therefore, the comparison of guidelines does not represent a complete analysis of all relevant guidelines. Furthermore, we assumed the completeness of the IMPP questions in the Viamedici online learning programme. A comparison with English textbooks and also the classification in the relevance of current research were cursory.

## Conclusions

Comparing the textbooks, Karow can be clearly distinguished from the other two textbooks. It is published annually and has more characteristics of a reference book. Despite its annual publication interval, the changes between editions are only small. Moreover, the strongest discrepancies with the hypertension guidelines are observed in this textbook. The current edition still mentions an indication for reserpine. Aside from that, drugs are mentioned in Karow that have not been on the market for longer time. Due to its publication interval, Karow suggests timeliness which is not necessarily true. Nevertheless, there are also advantages to Karow. It manages to fit a wealth of information clearly onto a few pages.

Aktories and Lüllmann are very similar in their conceptual structure. In contrast to Karow, they present a well worded text, often supported by diagrams. Reserpine tends to occur in several chapters, and the information is not as bundled as in the Karow. Neither textbook gives an indication for reserpine in the current edition. Lüllmann seems superior to Aktories in its conformity with guidelines on reserpine. The three textbooks cover many of the same topics, but each textbook has its own thematic focus that does not appear in the others. This can also be observed with the mentioned ADRs. Lüllmann is closer to guidelines in the topicality of the indication than Aktories and especially Karow. Reserpine is also not asked in IMPP questions. Nevertheless, the presence of reserpine in textbooks to support the explanation of mechanisms of action and for historical interest may still be justified. We also unmasked much more differences in the presentation of an “obsolete” drug in standard textbooks than anticipated. This renders it difficult to refer just to one textbook to obtain unequivocal information.

The relevance of reserpine in trials is very limited. Most likely, reserpine will disappear from pharmacology textbooks in the next decade. This development can be seen particularly well in Aktories. In the current edition, the chapter on reserpine is marked with a purple box and the heading “nur zur Vertiefung” (only for deepening knowledge).

Textbooks follow the text of their previous editions. This can leadto the fact that the entries and the presentation of reserpine are hardly ever revised, as seen in Karow. Overall, we showed that pharmacology textbooks, to different extents, lag behind current medical practice. It will be interesting to analyse to which extent the results on reserpine are transferable to other (largely) obsolete drugs such as phenylbutazone or indomethacin. Probing pharmacology textbooks for their coverage of obsolete drugs is a very informative way of assessing how up-to-date they are. 

## Supplementary information

ESM 1

## Data Availability

All source data for this study are available upon reasonable request.

## Abbreviations

ADR: Adverse drug reaction
VMAT2: Vesicular monoamine transporter 2
WIdO: Wissenschaftliches Institut der AOK (Scientific institute of the general local health insurance fund)
DDD: Defined daily dose
ATC: Anatomical - therapeutic - chemical classification
IMPP: Institut für medizinische und pharmazeutische Prüfungsfragen (Institute for medical and pharmaceutical exam questions)
