# Supplementary material for: Presentation of the obsolete drug reserpine in three German-language pharmacology textbooks
Source: Naunyn Schmiedebergs Arch Pharmacol. 2023 Dec 16;397(6):4381–401. doi: 10.1007/s00210-023-02877-9 (PMC11599303; doi:10.1007/s00210-023-02877-9)
Supplement: Supplementary file 1 — ESM 1 [file 210_2023_2877_MOESM1_ESM.pdf]

Supplemental data

Presentation of the "obsolete" drug Reserpine in German-language textbooks

Nikolas Misera, Roland Seifert

Supplement charts and data

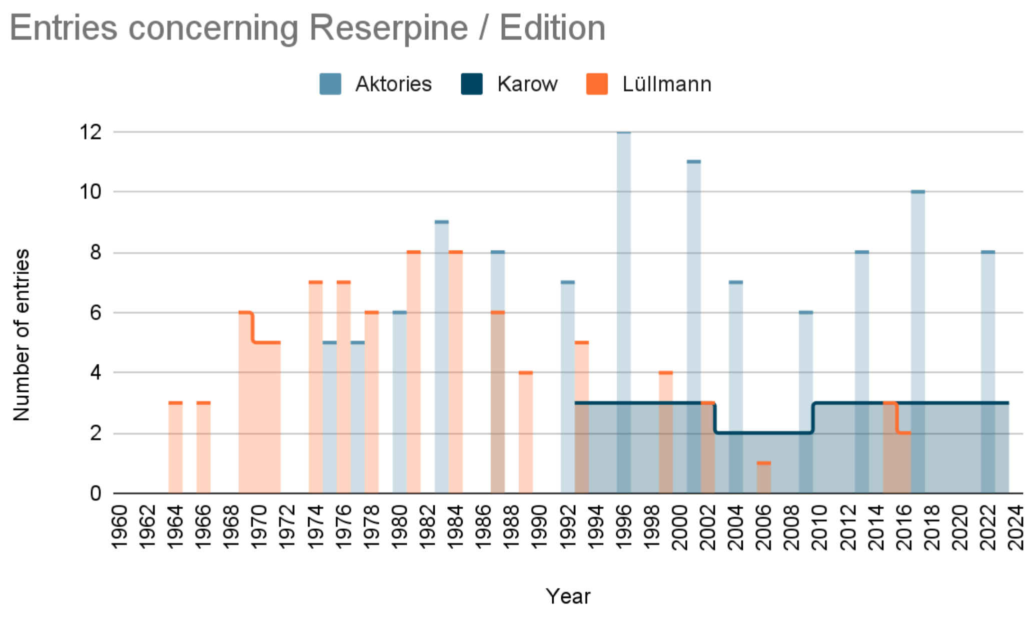

Figure S 1: The chart shows how many entries there are per edition in the keyword index regarding reserpine.

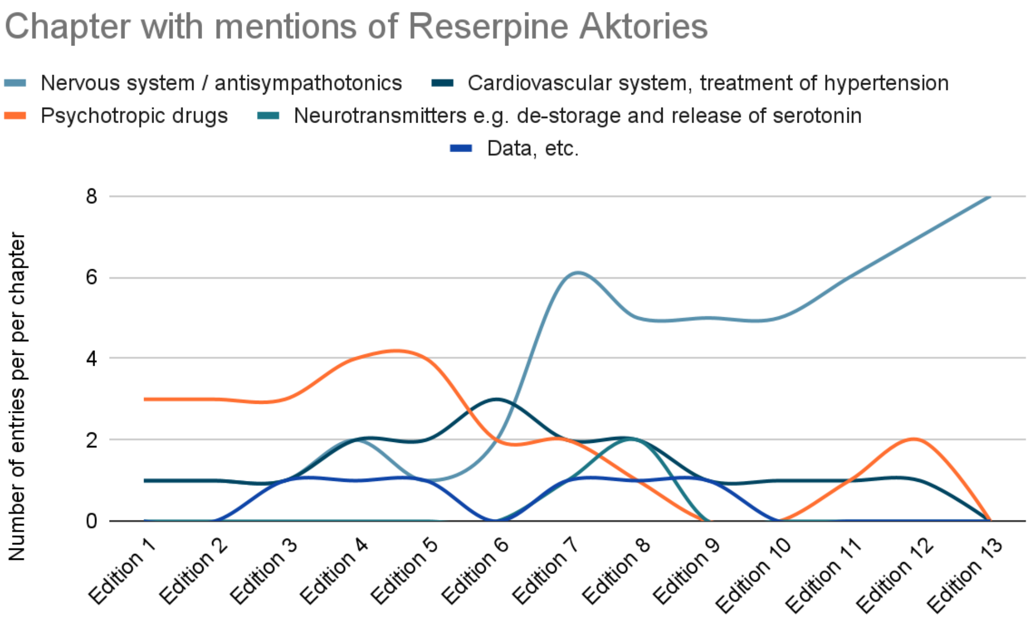

Figure S 2: The chart shows the mentions of reserpine in the different chapter groups in Aktories per edition.

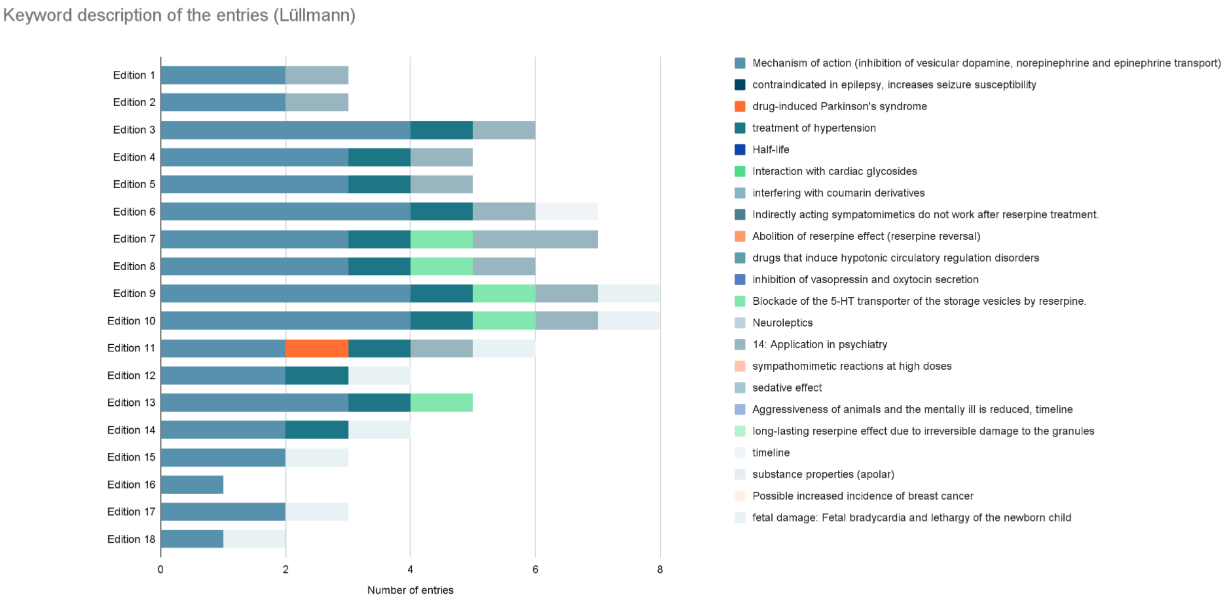

Figure S 3: The chart shows how many entries with a certain keyword (main topic) can be described per edition in Lüllmann.

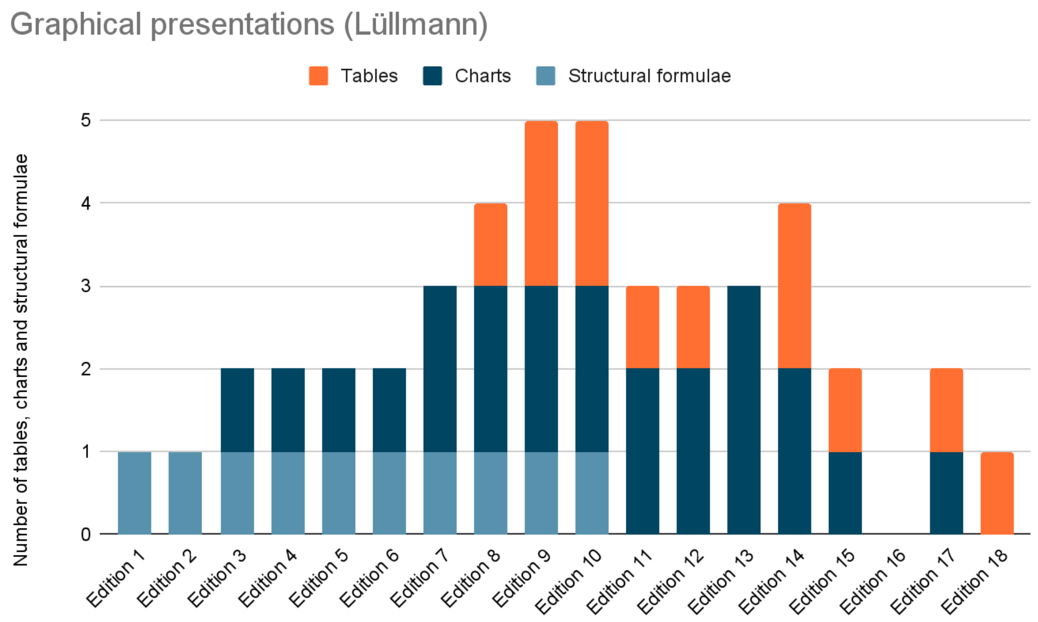

Figure S 4: The chart shows the number of graphical presentations per edition in Lüllmann. The graphical presentations were broken down into tables, diagrams and structural formulae.

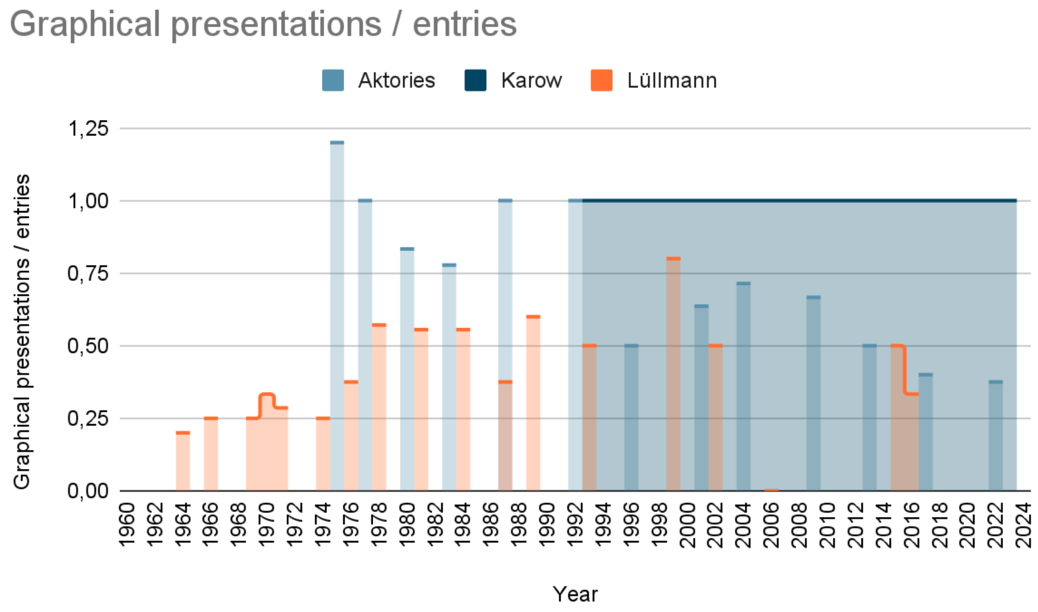

Figure S 5: The chart shows how many graphical presentations per number of entries occur in the three textbooks per edition.

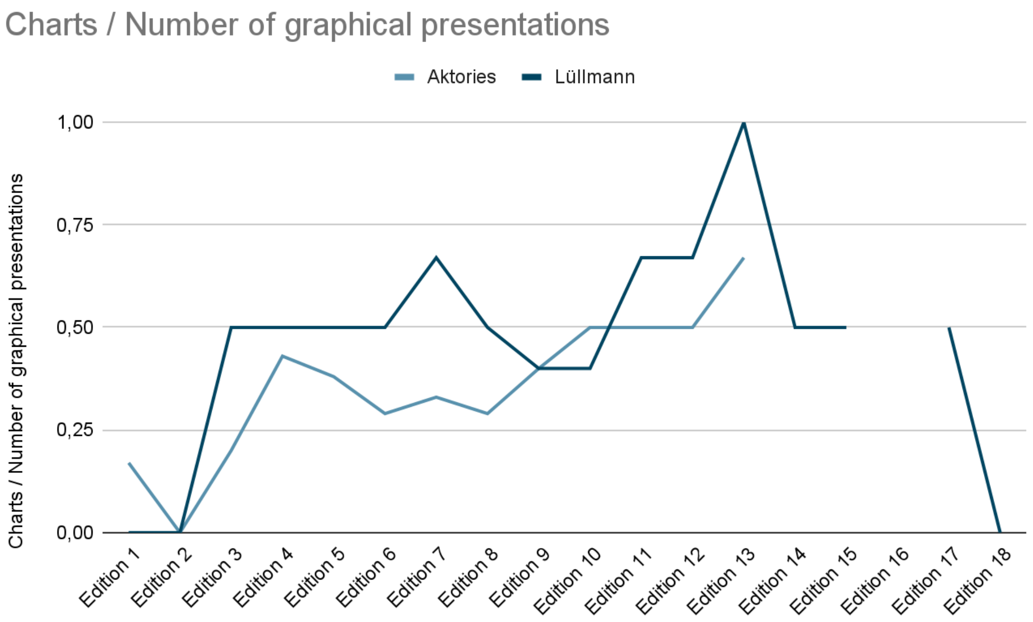

Figure S 6: The chart shows the share of charts among the graphical presentations in Lüllmann and Aktories per edition.

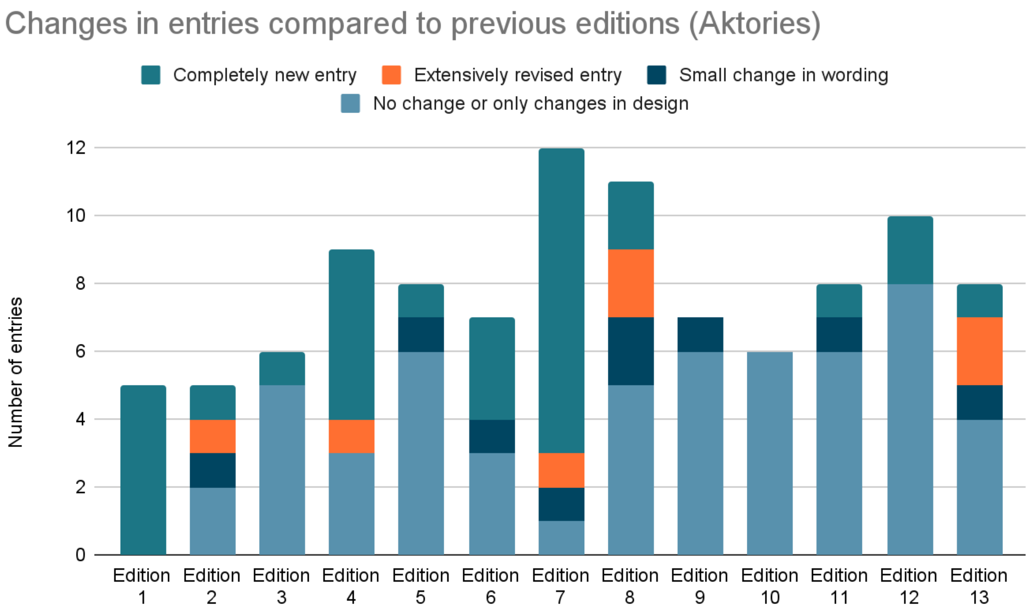

Figure S 7: The chart shows how much an entry changes compared to the previous edition. The chart shows the change per entry in Aktories.

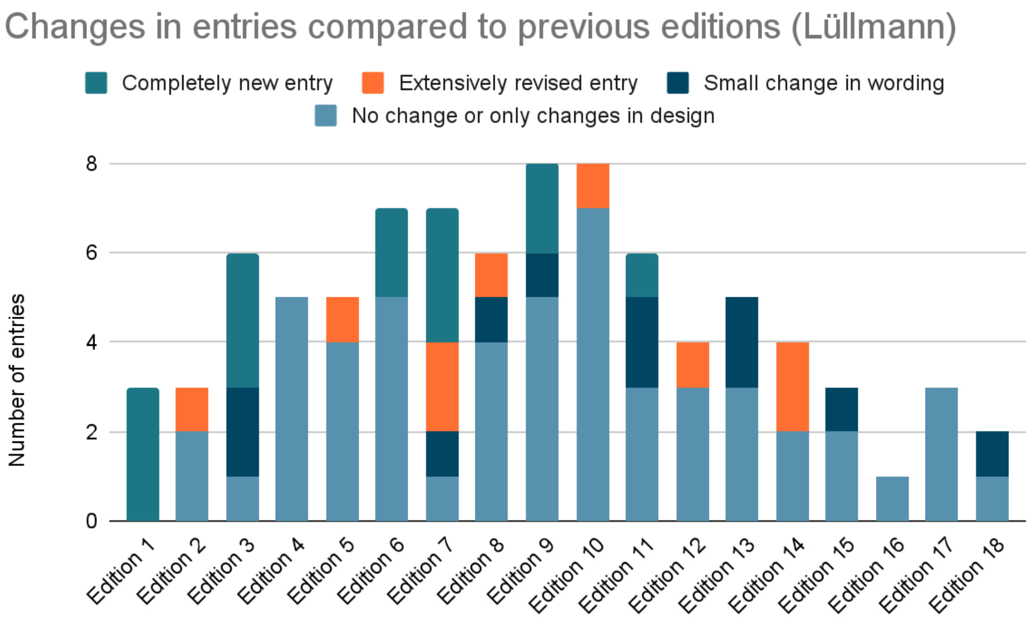

Figure S 8: The chart shows how much an entry changes compared to the previous edition. The chart shows the change per entry in Lüllmann.

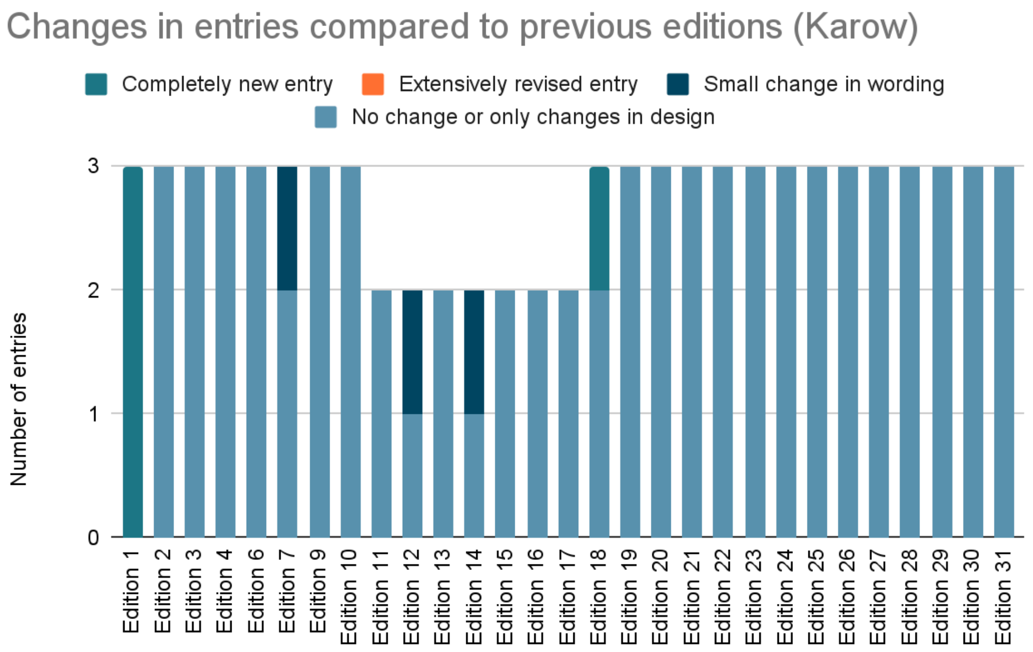

Chart S 9: The chart shows how much an entry changes compared to the previous edition. The chart shows the change per entry in Karow.

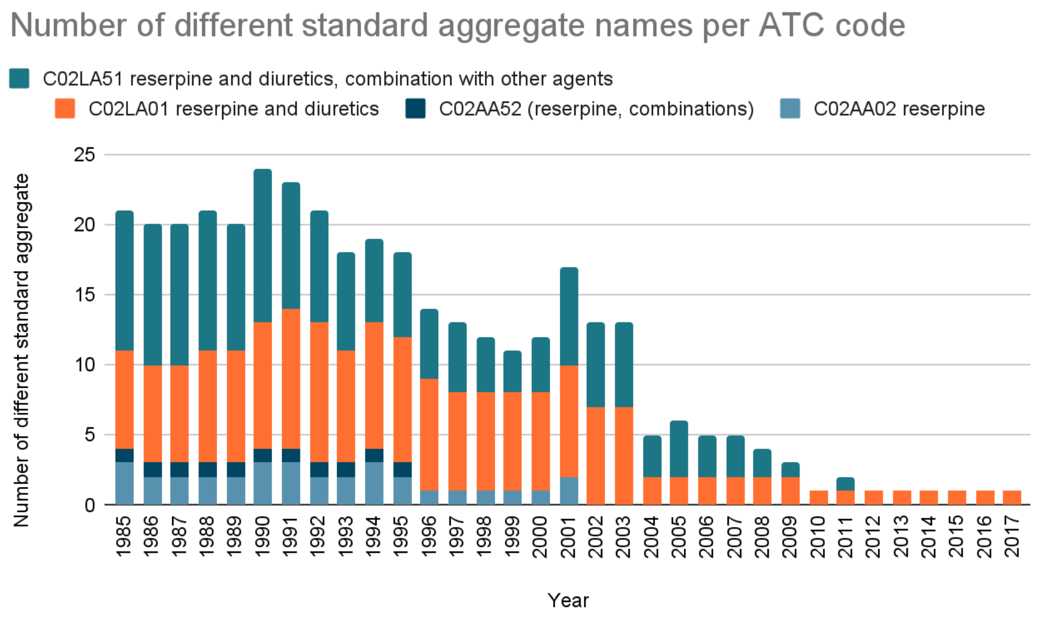

Figure S 10: The graph shows the number of standard aggregate names containing reserpine as an active ingredient per year. The data are broken down by ATC code.

**ATC Code**	**Standard aggregate name**	**Gross costs per DDD in Euro**
C02AA02 reserpine	Rausedan	0,1
	Reserpin Berco	0,1
	Reserpin Saar	0,2
	Serpasil	0,2
C02AA52 reserpine, combinations	Adelphan Tabl.	0,1
C02LA01 reserpine and diuretics	Disalpin	0,1
	Calmoserpin	0,2
	Reserpin Berco	0,2
	Durotan	0,2
	Modenol	0,2
	Barotonal	0,3
	Bendigon N	0,3
	Briserin N	0,3

	Terbolan	0,3
	Drenusil R	0,4
	Nortensin	0,4
	Darebon	0,5
C02LA51 reserpine and diuretics, combination with other agents	Triniton	0,1
	Repicin	0,2
	Tri.-Thiazid Reserpin STADA	0,2
	Adelphan-Esidrix	0,3
	Bendigon	0,3
	Briserin	0,3
	Caprinol	0,3
	Diuraupur	0,3
	Elfanex	0,3
	Resaltex	0,4
	Intensain 150-Modenol	0,8

Figure S 11: The table shows the gross costs per DDD in euros for all prescribed standard aggregate names from 1985 onwards. The standard aggregate names are broken down by ATC code.

Appendix 1: Dosage in the Karow:
Edition 12-31: The reserpine dose in the drug Briserin is 0.05 mg / 0.1 mg. The combination partner is clopamide at a dose of 2.5 mg / 5 mg. Maximum 3 doses / day
Other combination preparations contain reserpine + hydrochlorothiazide or chlortalidone, sometimes also dihydralazine.

Appendix 2: Dosage in Aktories:
1st edition: with a single administration of 0.25 mg reserpine, the elimination half-life is 271 hours.
2nd-3rd edition: no dosage stated
4th-5th edition: Reserpine is used in combined therapy with a contraindication to beta-receptor blockers in a daily dose of 0.1-0.3 mg. Basic therapy is given with a diuretic.
6th edition: no dosage mentioned

7th edition: adverse effects are low at the required doses, up to 0.25 mg / day. As a neuroleptic, reserpine was formerly used in doses up to 5 mg / day.
8th edition: Adverse effects are minor at the required doses, up to 0.25 mg / day. As a neuroleptic, reserpine was formerly used in doses up to 5 mg / day.
The adverse side effects (gastric intolerance up to ulcers, drowsiness and depression) were observed in doses of up to 0.75 mg / day applied in the 1960s and 1970s. Even doses of 0.1 mg / day can effectively lower blood pressure.
9th-12th edition: Adverse effects are low at the required doses, up to 0.25 mg / day. As a neuroleptic, reserpine was formerly used in doses up to 5 mg / day.
The adverse side effects (gastric intolerance up to ulcers, drowsiness and depression) were observed in doses of up to 0.75 mg / day used in the 1960s and 1970s. Even doses of 0.1 mg / day can effectively lower blood pressure (e.g. in Briserin with 5 mg clopamide).
13th edition: no dosage mentioned

Appendix 3: Dosage in Lüllmann:
1st-2nd edition: no dosage mentioned.
3rd-6th edition: reserpine can be used alone in daily doses of 0.25mg- 1mg or in combination with dihydralazine (30mg/day) in a daily dose of 0.3mg.
7th edition: Reserpine can be used alone in daily doses of 0.25mg- 1mg or in combination with dihydralazine (30mg/day) in a daily dose of 0.3 mg.
Even at a daily dose of 0.1-0.3 mg, a reduction in occupational performance must be expected.
At higher doses, the side effects increase. Reserpine then acts more like a neuroleptic.
The daily doses of reserpine are between 0.1-0.3 mg. As the effect is cumulative, a full effect can only be expected after a few weeks.
8th-9th edition: Even with a daily dose of 0.1-0.3 mg, a reduction in occupational performance must be expected.
At higher doses, the side effects increase. Reserpine then acts more like a neuroleptic.
The daily doses of reserpine are between 0.1-0.3 mg. As the effect is cumulative, a full effect can only be expected after a few weeks.
Reserpine should only be used for a short time at higher daily doses than 0.25 mg because of the risk of developing depression. The risk is considered low, but depression may persist long after discontinuation.
10th-11th edition: Even with a daily dose of 0.1-0.3 mg, a reduction in occupational performance must be expected.
At higher doses, the side effects increase. Reserpine then acts more like a neuroleptic.
The daily doses of reserpine are between 0.1-0.3 mg. As the effect is cumulative, a full effect can only be expected after several weeks.
12th edition: The daily doses of reserpine are between 0.1-0.3 mg. As the effect is cumulative, a full effect can only be expected after a few weeks.
13th-18th edition: no dosage mentioned

Appendix 4:
Briserin: Briserin contains the suffix -serin, this is very similar to the suffix -serpin of reserpine.
Sedaraupin: The name sedaraupin could be derived from the Latin word sedare, which can be translated as to calm or soothe, indicating its use in psychiatry.
Serpasil: The prefix Serp- could refer to the plant Rauwolfia serpentina, from which reserpine is obtained.
Modenol: Moderrere (Latin) means to tone down.
Tri-thiazide reserpine: The prefix tri- could refer to the 3 active ingredients hydrochlorothiazide, reserpine, triamterene. Thiazide alludes to the thiazide diuretics.
Darebon: "Dare" could come from the Latin word dare, which means to give or to present. Bon" could allude to a good effect.
Disalpin: The prefix dis- often has a meaning like remove (e.g. in disappear, disconnect) and could allude on the one hand to the removal of pain, but also to the reduction of catecholamines. alpi: Alps / mountains.
Durotan: The prefix Dur- (French) could suggest a strong effect.
Resaltex: The prefix could allude to the fact that the drug contains reserpine.

Appendix 5: Ranking of indications (hypertension).

1. *Application is not described:*
   ***13th edition (Aktories)**: Reserpine is of historical and experimental interest only. The chapter is marked "zur Vertiefung" (for deepening knowledge): Reserpine was one of the first effective agents for the treatment of hypertension. In addition, its mechanism of action has contributed greatly to the understanding of the biochemical basis of depression.*
2. *Obsolete, but still used:*
   ***18th edition (Lüllmann)**: Reserpine is obsolete nowadays, yet it is still used in fixed combination with a diuretic.*
   ***17th edition (Lüllmann)**: Reserpine is obsolete. Only in fixed combination with a diuretic is reserpine unfortunately still used.*
   ***16th edition (Lüllmann)**: Reserpine is obsolete. Only in fixed combination with a diuretic is reserpine unfortunately still used.*
   ***15th edition (Lüllmann)**: Reserpine is obsolete. Only in fixed combination with a diuretic is reserpine unfortunately still used.*
3. *Obsolete, but application is described in more detail:*
   ***14th edition (Lüllmann)**: Reserpine is obsolete, side effects are too severe compared to other antihypertensives. Only in fixed combination with a diuretic is reserpine still used quite frequently. A diagram shows the treatment plan for essential hypertension. Treatment with reserpine is in the 3rd stage in a three-drug combination + a substance from the 2nd stage (beta blocker, ACE inhibitor or calcium antagonist).*
4. *Reserpine is a drug of further choice, the side effects are described as very severe:*
   ***7th edition (Aktories)**: Due to the side effect, reserpine is considered a second-choice antihypertensive, but can be used well as a combination partner if therapy with a first-choice antihypertensive is not sufficient.*

Reserpine is now of little importance in the treatment of hypertension because of its side effects. The side effects limit the patients' quality of life too much.
1st-17th edition (Karow)*: The indication for reserpine is the combination therapy of hypertension. Due to the side effects, however, it is only a drug of distant choice. In edition 7-13, before the chapter on reserpine, it is written that reserpine is relevant to the examination, but has only a minor therapeutic significance.*

5. *The side effects described in the past are based on too high doses. The advantages of reserpine are described, but no more detailed application is explained:*
8th edition (Aktories)*: The side effects mentioned were observed in the 1960s and 1970s at daily doses of 0.75 mg. Due to the long half-life, a daily dose of 0.1 mg can already effectively lower blood pressure. At this dosage, side effects are rare. The low therapy costs are mentioned as an advantage over other antihypertensives.*
9th edition (Aktories)*: The side effects mentioned were observed in the 1960s and 1970s at daily doses of 0.75 mg. Due to the long half-life, a daily dose of 0.1 mg can already effectively lower blood pressure. At this dosage, side effects are rare. The low therapy costs are mentioned as an advantage over other antihypertensives. In addition to the previous edition, it is written here that the use of reserpine in the treatment of hypertension is only marginal.*
10th edition (Aktories)*: The side effects mentioned were observed in the 1960s and 1970s at daily doses of 0.75 mg. Due to the long half-life, a daily dose of 0.1 mg can already effectively lower blood pressure. At this dosage, side effects are rare. The low therapy costs are mentioned as an advantage over other antihypertensives. The use of reserpine in the treatment of hypertension is only marginal.*
11th edition (Aktories)*: The side effects mentioned were observed in the 1960s and 1970s at daily doses of 0.75 mg. Due to the long half-life, a daily dose of 0.1 mg can already effectively lower blood pressure. At this dosage, side effects are rare. The low therapy costs are mentioned as an advantage over other antihypertensives. The use of reserpine in the treatment of hypertension is only marginal.*
12th edition (Aktories)*: The side effects mentioned were observed in the 1960s and 1970s at daily doses of 0.75 mg. Due to the long half-life, a daily dose of 0.1 mg can already effectively lower blood pressure. At this dosage, side effects are rare. The low therapy costs are mentioned as an advantage over other antihypertensives. The use of reserpine in the treatment of hypertension is only marginal.*
6. *Indication for therapy-refractory hypertension, severe forms of hypertension and in case of existing contraindication for beta-blockers. Therapeutic applicability is limited to severe, otherwise resistant forms of hypertension due to side effects:*
12th edition (Lüllmann)*: If there is a contraindication to hypertension treatment with saluretics and beta-blockers, or if the hypertension is too*

severe, therapy with an antisympathotonic such as reserpine can be resorted to. In therapy-refractory hypertension, either the dose of antihypertensives must be increased or the combination partners should be exchanged for stronger ones such as reserpine.
In a table, reserpine and a saluretic are mentioned as options for the therapy of severe hypertension. In addition to the 11th edition, it is mentioned here that hypertension treatment is limited only to severe, otherwise resistant forms of hypertension.
***13th edition (Lüllmann)**: If there is a contraindication regarding hypertension treatment with saluretics and beta-blockers, or if the hypertension is too severe, therapy with an antisympathotonic drug such as reserpine can be resorted to. In therapy-refractory hypertension, either the dose of antihypertensives must be increased or the combination partners should be exchanged for stronger ones such as reserpine.*
In a table, reserpine and a saluretic are mentioned as options for the therapy of severe hypertension. In addition to the 11th edition, it is mentioned here that hypertension treatment is limited only to severe, otherwise resistant forms of hypertension.
***18th-31st edition (Karow)**: The indication for reserpine is mentioned as combination therapy of hypertension. Due to the side effects, however, it is only a drug of distant choice. Therapy-refractory hypertension is mentioned as an indication in a table.*

7. *Indication for therapy-refractory hypertension, severe forms of hypertension and in case of existing contraindication for beta-blockers. The therapeutic usability is clearly limited due to the side effects:*
   ***11th edition (Lüllmann)**: If there is a contraindication with regard to hypertension treatment with saluretics and beta-blockers or the hypertension is too severe, therapy with an antisympathotonic such as reserpine can be resorted to. In therapy-refractory hypertension, either the dose of antihypertensives must be increased or the combination partners should be exchanged for stronger ones such as reserpine.*
   In a table, reserpine and a saluretic are mentioned as options for the therapy of severe hypertension (compared to the 10th edition: here moderate hypertension is mentioned).
8. *Indication for therapy-refractory hypertension, moderately severe forms of hypertension and in case of existing contraindication for beta-blockers. The therapeutic usability is clearly limited due to the side effects:*
   ***10th edition (Lüllmann)**: If there is a contraindication regarding hypertension treatment with saluretics and beta-blockers or the hypertension is too severe, therapy with an antisympathotonic such as reserpine can be resorted to. In therapy-refractory hypertension, either the dose of antihypertensives must be increased or the combination partners should be exchanged for stronger ones such as reserpine.*
   A table lists reserpine and a saluretic as options for the treatment of moderate hypertension.
   ***4th edition (Aktories)**: Due to the side effect, the therapeutic use of reserpine is restricted.*

The indication for reserpine is the treatment of hypertension in cases where beta-receptor blockers are contraindicated. For basic therapy, a diuretic is used here and clonidine and prazosin as additives. Reserpine is also mentioned as the antihypertensive of further choice.
5th edition (Aktories): *Due to the side effect, the therapeutic use of reserpine is restricted.*
The indication for reserpine is the treatment of hypertension in cases where beta-receptor blockers are contraindicated. For basic therapy, a diuretic is used here and clonidine and prazosin as additives. Reserpine is also mentioned as the antihypertensive of further choice.
6th edition (Aktories)*: Due to the side effect, the therapeutic use of reserpine is restricted.*
The indication for reserpine here is the treatment of hypertension in cases where beta-receptor blockers are contraindicated. For basic therapy, a diuretic is used here and clonidine and prazosin as additives. Reserpine is not considered a first-choice antihypertensive.

9. *<u>Due to the side effects, an indication is only given for severe forms of hypertension:</u>*
   8th edition (Lüllmann)*: Reserpine is described as having good efficacy but, because of the risk of developing depression, should only be used for short periods at higher daily doses than 0.25mg. The risk of this is considered low, but depression can occur long after discontinuation. Only severe forms of hypertension are mentioned in the indication.*
   9th edition (Lüllmann)*: Reserpine is described as having good efficacy but, because of the risk of developing depression, should only be used for short periods at daily doses higher than 0.25mg. The risk of this is considered low, but depression can occur long after discontinuation. Only severe forms of hypertension are mentioned in the indication.*
10. *<u>Indication for hypertension treatment, the adverse side effects are weighted more heavily:</u>*
   7th edition (Lüllmann)*: The side effects, after small doses of reserpine, are described here as tolerable. However, a reduction in performance must already be expected (at doses of 0.1-0.3 mg / day). To keep the side effects low, reserpine should be used in combination with other antihypertensives. Reserpine can be used if hypertension is not adequately treated by a low-salt diet and the administration of saluretics. The effect of reserpine is reported to be good.*
   1st edition (Aktories)*: A factual list of side effects is given. No recommendation is made, but neither is it stated that reserpine is obsolete.*
   2nd edition (Aktories)*: As in the first edition, a factual list of side effects is given. No recommendation is made, but neither is it stated that reserpine is obsolete. Another disadvantage mentioned is the slow onset of action.*
   3rd edition (Aktories)*: As in the first edition, a factual list of side effects is given. No recommendation is made, but neither is it stated that reserpine is obsolete. Another disadvantage mentioned is the slow onset of action.*
11. *<u>Indication for hypertension treatment. Reserpine is described as having good efficacy:</u>*

***1st edition (Lüllmann)**: Reserpine is described as an essential agent in hypertension therapy.*
***2nd edition (Lüllmann)**: Reserpine is used for hypertension treatment, but it is not explicitly described here as an essential agent in hypertension therapy compared to the 1st edition.*
***3rd edition (Lüllmann)**: In an additional chapter on hypertension treatment, it is described that reserpine can be resorted to if hypertension is insufficiently treated by a low-salt diet and the administration of saluretics. The effect of reserpine is described as good.*
***4th edition (Lüllmann)**: In an additional chapter on hypertension treatment, it is described that reserpine can be resorted to if hypertension is inadequately treated by a low-salt diet and the administration of saluretics. The effect of reserpine is described as good.*
***5th edition (Lüllmann)**: In an additional chapter on hypertension treatment, it is described that reserpine can be resorted to if hypertension is insufficiently treated by a low-salt diet and the administration of saluretics. The effect of reserpine is described as good.*
***6th edition (Lüllmann)**: In an additional chapter on hypertension treatment, it is described that reserpine can be resorted to if hypertension is insufficiently treated by a low-salt diet and the administration of saluretics. The effect of reserpine is described as good.*

Appendix 6: Ranking of indications (psychoses)

0. *<u>No mention of reserpine for the treatment of psychoses:</u>*
   ***12th -18th edition (Lüllmann):** no mention of reserpine for the treatment of psychoses*
   ***1st-31st edition (Karow)**: no mention of reserpine in the treatment of psychoses.*
   ***1st-3rd edition (Aktories):** no mention of reserpine for the treatment of psychoses.*
   ***5th-13th edition (Aktories)**: no mention of reserpine for the treatment of psychoses.*
1. *<u>Reserpine was used in the past in psychiatry. The indication was abandoned due to the strong side effects:</u>*
   ***7th edition (Lüllmann)**: In addition to its main field of application, the treatment of hypertension, it was used for anxiety and stress states as well as chronic psychoses with psychomotor hyperactivity and aggressiveness because of its sedative and neuroleptic effects. Because of too strong side effects occurring at the high doses required for this purpose, this indication for reserpine has been abandoned.*
   ***9th edition (Lüllmann):** In addition to its main field of application, the hypertension treatment, it was used for anxiety and stress states and chronic psychoses with psychomotor hyperactivity and aggressiveness because of its sedative and neuroleptic effects. Because of too strong side*

effects occurring at the high doses required for this purpose, this indication for reserpine has been abandoned.
10th edition (Lüllmann): *In addition to its main field of application, the hypertension treatment, it was used for anxiety and stress states and chronic psychoses with psychomotor hyperactivity and aggressiveness because of its sedative and neuroleptic effects. Because of too strong side effects occurring at the high doses required for this purpose, this indication for reserpine has been abandoned.*
11th edition (Lüllmann): *In addition to its main field of application, the hypertension treatment, it was used for anxiety and stress states and chronic psychoses with psychomotor hyperactivity and aggressiveness because of its sedative and neuroleptic effects. Because of too strong side effects occurring at the high doses required for this purpose, this indication for reserpine has been abandoned.*

2. *<u>Reserpine was used in the past in psychiatry. However, the reason (strong ADRs) is not mentioned:</u>*
5th edition (Lüllmann): *In addition to its main field of application, the treatment of hypertension, it was used for its sedative and neuroleptic effects in states of anxiety and stress as well as chronic psychoses with psychomotor hyperactivity and aggressiveness.*
6th edition (Lüllmann): *In addition to its main field of application, the treatment of hypertension, it was used for its sedative and neuroleptic effects in states of anxiety and stress as well as chronic psychoses with psychomotor hyperactivity and aggressiveness.*
8th edition (Lüllmann): *In addition to its main field of application, the treatment of hypertension, it was used for its sedative and neuroleptic effects in states of anxiety and stress as well as chronic psychoses with psychomotor hyperactivity and aggressiveness.*
3. *<u>Reserpine is now only rarely used in psychiatry:</u>*
4th edition (Aktories): *The neuroleptic reserpine is now rarely used in psychiatry.*
4. *<u>There is an indication for the treatment of psychoses with reserpine. However, other drugs are preferred:</u>*
1st edition (Lüllmann)*: For the indication treatment of psychoses: In addition to the main field of application, the treatment of hypertension, reserpine is used for anxiety and stress states as well as chronic psychoses with psychomotor hyperactivity and aggressiveness, due to its sedative and neuroleptic effect. In the treatment of acute psychoses, agents from the chlorpromazine group are preferred.*
2nd edition (Lüllmann): *For the indication treatment of psychoses: In addition to the main field of application, the treatment of hypertension, reserpine is used for anxiety and stress states as well as chronic psychoses with psychomotor hyperactivity and aggressiveness, due to its sedative and neuroleptic effect. In the treatment of acute psychoses, agents from the chlorpromazine group are preferred.*
3rd edition (Lüllmann): *For the indication treatment of psychoses: In addition to the main field of application, the treatment of hypertension,*

reserpine is used for anxiety and stress states as well as chronic psychoses with psychomotor hyperactivity and aggressiveness, due to its sedative and neuroleptic effect. In the treatment of acute psychoses, other neuroleptics are usually preferred.

4th edition (Lüllmann): *For the indication treatment of psychoses: In addition to the main field of application, the treatment of hypertension, reserpine is used for anxiety and stress states as well as chronic psychoses with psychomotor hyperactivity and aggressiveness, due to its sedative and neuroleptic effect. In the treatment of acute psychoses, other neuroleptics are usually preferred.*
